# Insights into the physico-chemical and biological characterization of sodium lignosulfonate - silver nanosystems designed for wound management

**DOI:** 10.1016/j.heliyon.2024.e26047

**Published:** 2024-02-09

**Authors:** Ioana C. Marinas, Leonard Ignat, Ignat E. Maurușa, Madalina D. Gaboreanu, Coroabă Adina, Marcela Popa, Mariana C. Chifiriuc, Marian Angheloiu, Mihaela Georgescu, Alexandra Iacobescu, Gratiela Gradisteanu Pircalabioru, Miruna Stan, Mariana Pinteala

**Affiliations:** a Research Institute of the University of Bucharest—ICUB, University of Bucharest, 050095, Bucharest, Romania; b Research and Development Department of SC Sanimed International Impex SRL, 6 Bucharest -Giurgiu Street, 087040, Giurgiu, Romania; c Centre of Advanced Research in Bionanoconjugates and Biopolymers, “Petru Poni” Institute of Macromolecular Chemistry, Gr. Ghica Voda Alley 41A, Iasi, 700487, Romania; d Faculty of Biology, Department of Botany and Microbiology, University of Bucharest, 1-3 Portocalelor Street, 060101, Bucharest, Romania; e Romanian Academy of Scientists, 54 Spl. Independentei St., District 5, 50085, Bucharest, Romania; f The Romanian Academy, 25, Calea Victoriei, Sector 1, District 1, 010071, Bucharest, Romania; g Faculty of Biology, Department of Biochemistry and Molecular Biology, University of Bucharest, 91-95 Splaiul Independentei, 050095, Bucharest, Romania

**Keywords:** Sodium lignosulfonate, Silver nanoparticle, Green synthesis, Antimicrobial activity, Nitric oxide, Hemocompatibility

## Abstract

Chronic wounds represent one of the complications that might occur from the disruption of wound healing process. Recently, there has been a rise in interest in employing nanotechnology to develop novel strategies for accelerating wound healing. The aim of the present study was to use a green synthesis method to obtain AgNPs/NaLS systems useful for wounds management and perform an in-depth investigation of their behavior during and post-synthesis as well as of their biological properties. The colloids obtained from silver nanoparticles (AgNPs) and commercial sodium lignosulfonate (NaLS) in a single-pot aqueous procedure have been fully characterized by UV–Vis, FT-IR, DLS, TEM, XRD, and XPS to evaluate the synthesis efficiency and to provide new insights in the process of AgNPs formation and NaLS behavior in aqueous solutions. The effects of various concentrations of NaLS (0–16 mg/mL) and AgNO_3_ (0–20 mM) and of two different temperatures on AgNPs formation have been analyzed. Although the room temperature is feasible for AgNPs synthesis, the short mixing at 70 °C significantly increases the speed of nanoparticle formation and storage stability. In all experimental conditions AgNPs of 20–40 nm in size have been obtained. The antimicrobial activity assessed quantitatively on clinical and reference bacterial strains, both in suspension and biofilm growth state, revealed a broad antimicrobial spectrum, the most intensive inhibitory effect being noticed against *Pseudomonas aeruginosa* and *Escherichia coli* strains. The AgNP/NaLS enhanced the NO extracellular release, potentially contributing to the microbicidal and anti-adherence activity by protein oxidation. Both AgNP/NaLS and NaLS were non-hemolytic (hemolytic index<5%, 2.26 ± 0.13% hemolysis) and biocompatible (102.17 ± 3.43 % HaCaT cells viability). The presence of AgNPs increased the antioxidative activity and induced a significant cytotoxicity on non-melanoma skin cancer cells (62.86 ± 8.27% Cal-27 cells viability). Taken together, all these features suggest the multivalent potential of these colloids for the development of novel strategies for wound management, acting by preventing infection-associated complications and supporting the tissue regeneration.

## Graphical abstract

**Figure:**
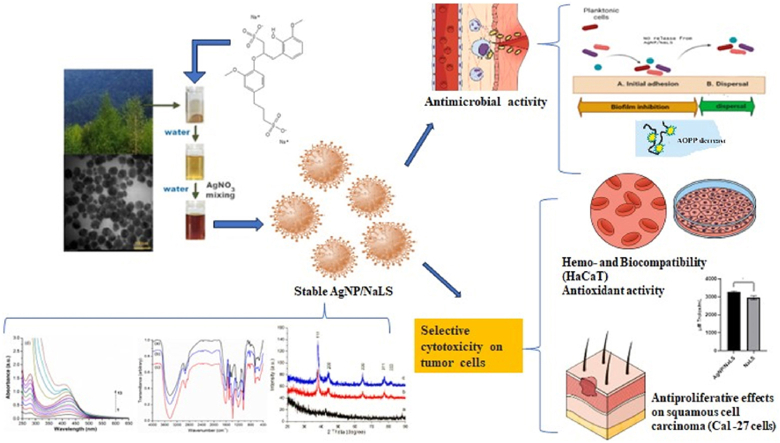

## 1 Introduction

Even though from environmental and/or healthcare point of view nanosized materials may become themselves hazardous in certain situations [[1], [2], [3]], their specific size-dependent properties remain critical for newly emerging key technologies in a wide range of domains, including green chemistry [[4], [5], [6]]. In order to reduce the potential risks associated to their expanding applications, the principles of green chemistry [7] are more and more applied in synthesis, leading to a continuous increase and diversification of environmental-friendly nanomaterials and processes.

Silver nanoparticles (AgNPs) are among the first and most extensively studied materials at the nanoscale, being used in various applications, long before the advent of nanotechnology [8]. Their historical uses, as well as the renewed interest shown by the current widespread research reports and commercial products, were mainly triggered by their remarkable antimicrobial properties [9,10], covering areas like medicine, cosmetics, and packaging [[11], [12], [13]].

The continuous development of characterization tools and techniques at nanoscale level has pointed out the optical, chemical, and conductive properties of AgNPs [14]. Besides common antimicrobial uses, these highly specific and valuable characteristics of AgNPs are currently fueling a wide range of applications, from catalysis [15], biosensors [16], biolabeling [17] and bioassays [18] to photovoltaics [19] and enhanced optical spectroscopies [20].

The AgNPs properties and applications strictly depend on their size, shape, polydispersity and stability, which are in turn conditioned by the synthesis parameters like silver salts concentration, type of reducing and stabilization agents, reaction media, time, and temperature [[21], [22], [23]]. These reactions generally require hazardous chemicals, highly active compounds or synthetic polymers; in a typical synthesis, silver nitrate is reduced in organic solvents or aqueous media by a potent reducing agent (e.g., sodium borohydride) at low temperatures or by a milder one (e.g., sodium citrate) at boiling, whereas the resulting nanoparticles are stabilized by capping with polymers like poly (N-vinyl-2-pyrrolidone) or specific low molecular weight ligands (i.e., alkane thiolates) [[24], [25], [26]]. Time and energy consuming operations are additionally needed for both product purification and containment of residual pollutants.

The continuous increase of human society awareness regarding the environmental impact attracts numerous attempts to harmonize as much as possible the production of chemicals, including metal nanoparticles synthesis, with the green chemistry principles [27]. A tremendous number of reports popped up in the last decade claiming various green alternatives for AgNPs synthesis, based either on biosynthesis by microorganisms [[28], [29], [30]] and plants [31], or on using a wide range of biomass fractions and derivatives as substitutes for traditionally employed harsh chemicals. However, most of them involve quite laborious or time-consuming operations, while the final complex mixtures and products characteristics in terms of stability, size, shape, and polydispersity are difficult to manage.

These drawbacks were partially overcome when aqueous extracts from specific plant components like leaves or fruits were used for both metal salt reduction and nanoparticle capping [32,33]. Such extracts usually contain a complex mixture of numerous biological compounds, but their systematic analysis strongly suggests that polysaccharides [34,35] and polyphenols [[36], [37], [38]] are ones of the main active species responsible for nanoparticle formation and stabilization. Although the reduction of silver ions may be also attained with low molecular weight phenols and monosaccharides [39,40], the capping performances are considered as limited comparing with polysaccharides [[41], [42], [43]] and polymer-type polyphenols like tannins [44] and humic acids [45].

Despite the large variety of polyphenol-rich vegetal extracts used to synthesize AgNPs, the potential of corresponding plant-derived polymers, lignin and its natural or processed derivatives like humic acids and lignosulphonates, was lesser and rather superficially investigated. Humic acids, which naturally result through the environmental degradation of lignin as a mixture of polymers with multiple phenolic and carboxylic functional groups, showed good capping properties when analyzed as green substitutes in AgNPs syntheses [45,46].

The water-soluble lignosulphonates have been also considered as potential candidates to synthesize stabilized silver, platinum, or palladium nanoparticles in highly alkaline media [46,47]. The most common is sodium lignosulphonate (NaLS), which could be recovered in large amounts from the spent pulping liqueurs as a main by-product of the pulp and paper industry [48]. Notwithstanding a usual high polydispersity, is a readily available, cost-effective commercial material characterized by low environmental impact and high-water solubility, which is more homogeneous and easier to retrieve then humic compounds [49,50]. Furthermore, its distinctive structural features, related to the combination of a complex hydrophobic three-dimensional backbone composed from randomly interconnected phenolic and phenylpropanoid units and a significant amount of hydrophilic sulfonic groups, are potentially valuable for nanoparticle synthesis [49,50]. For example, NaLS macromolecules exhibit oblate conformations and negatively charged surfaces, with tendencies of self-assembly and loosely aggregate in water [[51], [52], [53]]. Thus, NaLS not only triggers the formation and stabilization of AgNPs, but it could further protect them in either physico-chemical or biological aggressive environments, mediates their attachment/confinement on surfaces, tunes the release of silver ions, and allows the design of complex material, extending the applicative field [[54], [55], [56]]. In this regard, we have previously reported, probably for the first time, the use of NaLS for the reduction and stabilization of silver ions, in the absence of any additional process, as one of the simplest, facile, cost-efficient, and greener synthesis for obtaining stable and catalytically active AgNPs [56,57], which was later confirmed by works reported by other groups [58,59]. On the other hand, due to their intrinsic structural and functional properties and especially to a large spectrum of antimicrobial activity, AgNPs are still in the focus for the development of novel strategies to control wound infections and promote the healing process [60]. AgNPs have been also proved to exhibit immunomodulatory properties, which may enhance their antibacterial effect by regulating the intensity of the inflammatory processes and thus promote tissue regeneration [61]. Considering all these aspects, our aim was to perform an in-depth investigation of the AgNPs/NaLS systems behavior during and post-synthesis as well as of their biological properties that could support their potential use for wound management.

## 2 Materials and methods

### 2.1 Materials

Silver nitrate (AgNO_3_, 99%) was purchased from Sigma-Aldrich and sodium lignosulfonate (NaLS) from Carl Roth (Karlsruhe, Germany). NaLS was provided under the form of a fine, homogeneous brown powder (>93% dry substance) with good water solubility (pH∼8.5), a molecular weight of 18100 g/mol and a polydispersity index of 2.225. Both AgNO_3_ and NaLS were used as received, without any further purification. Ultrapure deionized water was used in all instances.

### 2.2 Synthesis of AgNPs by NaLS

Two stock solutions of NaLS (2 mg/mL) and respective AgNO_3_ (0.1 M) were prepared with deionized water and well homogenized prior to the nanoparticle's synthesis. The reaction mixtures were adjusted with deionized water to reach final concentrations of 1.6 mg/mL NaLS and 1.6 mM AgNO_3_ and incubated under stirring (600 rpm) for either 24 h at room temperature or 2 h at 70 °C. pH was maintained neutral during the onset of reaction by dropping NaOH (0.1 M). The resulting colloidal solutions were maintained for 2 weeks at room temperature under occasional shaking to evaluate the short-term evolution of AgNPs. Alternatively, the room temperature desiccation under vacuum led to black grains with crystalline appearance that could be used to restore the AgNPs/NaLS aqueous colloids when needed.

To evaluate the effect of concentration of each reaction component, two types of mixtures containing either (i) the same AgNO_3_ concentration (1.6 mM) and NaLS amounts ranging from 0 to 16 mg/mL, or (ii) various AgNO_3_ concentrations (0–20 mM) and 1.6 mg/mL fixed NaLS content (Table 1), were reacted under shaking conditions (600 rpm) in borosilicate test tubes placed in an Eppendorf® thermomixer. All solutions were pale yellowish to dark brown depending on the initial concentration of reactants. The other experimental conditions remained unchanged. All reactions were carried out in triplicate, with freshly prepared solutions.

**Table 1:** AgNO_3_ and NaLS concentrations used in the AgNPs synthesis.

Sp.	AgNO_3_ (mM)	NaLS (mg/mL)	Sp.	AgNO_3_ (mM)	NaLS (mg/mL)	Sp.	AgNO_3_ (mM)	NaLS (mg/mL)
1	0	1.6	1	1.6	0.32	9	1.6	2.88
2	0.4	1.6	2	1.6	0.64	10	1.6	6.4
3	0.8	1.6	3	1.6	0.96	11	1.6	9.6
4	2.0	1.6	4	1.6	1.28	12	1.6	12.8
5	4.0	1.6	5	1.6	1.6	13	1.6	16
6	8.0	1.6	6	1.6	1.92	–	–	–
7	12.0	1.6	7	1.6	2.24	–	–	–
8	20.0	1.6	8	1.6	2.56	–	–	–

### 2.3 AgNPs characterization

The synthesized AgNPs were characterized without any additional purification of the resulting AgNPs/NaLS colloidal solutions. The AgNPs formation and stability were examined with a UV–Vis spectrophotometer (Perkin Elmer LAMBDA 35, USA) by scanning the wavelength range of 250 nm–650 nm. All samples have been diluted 10-fold or 20-fold to obtain an appropriate scale-up of the relative absorbance readings before analysis in standard, 1 cm optical path quartz cuvettes (Hellma, Germany). Ultrapure deionized water was used for dilution and as a blank.

Zeta potential and dynamic light scattering (DLS) measurements were carried out at 25 °C on pristine AgNPs/NaLS solutions, by using a Delsa Nano C submicron particle size analyzer (Beckman Coulter, UK), to estimate the surface charge and size distribution of colloidal nanoparticles. The distribution curves were plotted based on Stokes-Einstein equation, which assumes the presence of monodisperse particles.

The nanoparticle morphologies were observed by a transmission electron microscope (TEM; Hitachi HT 7700, Japan) in high resolution mode, at 120 KV, and a field emission scanning electronic microscope (FESEM; Verios G4UC, Thermo Scientific, Brno, Czech Republic) equipped with a STEM3+ detector at an accelerating voltage of 25 kV. Samples were 10-fold diluted with deionized water, homogenized, drop-casted on carbon-coated grids and dried at room temperature.

The elemental analysis of AgNPs/NaLS mixture was performed by X-ray photoelectron spectroscopy (XPS; Kratos Analytical Axis Nova, UK) on desiccated samples obtained from drop-casting of undiluted solutions. The incident monochromatic X-ray beam (1486.6 eV AlKα radiation, source of 300 W at 15 kV) was focused on a central area of 0.7 mm × 0.3 mm from the shell surface of samples. The XPS survey spectra were collected in 1eV steps at 160eV pass energy, whereas high-resolution core spectra of individual chemical elements were acquired at 20eV pass energy, with a resolution of 0.1eV. The binding energy of the C 1s peak was normalized to 285.0 eV and used as charge reference. Linear background subtractions were made before the correction of core peak areas. Data analysis and spectra fitting were done by using the Kratos original processing software Vision 2.2.8. The structural analysis was furthermore conducted on a Fourier transform infrared spectrometer (FTIR; Bruker Optics Vertex 70, Germany). The liquid samples were concentrated and dried under vacuum at room temperature to obtain fine-grained powders suitable for analysis. Spectra were recorded over 600-4000 cm^−1^ domain (64 scans) using the attenuated total reflectance technique (ATR-FTIR) and Bruker Opus 5 software.

The crystallinity of AgNPs was evaluated with a Bruker-AXS D8 ADVANCE X-ray diffractometer (XRD) using a Ni-filtered CuKα radiation (k = 0.1541 nm) and setting a 40 kV and 25 mA tube power working conditions. The fine powdered samples were prepared through advanced desiccation of NaLS and AgNPs/NaLS solutions, followed by milling. All diffraction patterns were acquired at room temperature in the range of 20–90° 2θ degrees, in step of 0.0200.

### 2.4 Antimicrobial activity

#### 2.4.1 Qualitative evaluation of the antimicrobial activity

The qualitative screening of the antimicrobial activity was performed by an adapted diffusion method on Muller Hinton agar medium inoculated with standard bacterial cell suspensions prepared from reference strains traceable to ATCC (*Staphyloccocus aureus* ATCC 25923, *Enterococcus faecalis* ATCC 29212, *Pseudomonas aeruginosa* ATCC 27853, *Escherichia coli* ATCC 25922) and clinical strains, isolated from wound infections and included in the microbial collection of the Faculty of Biology, University of Bucharest, i.e. methicillin resistant *S. aureus* (MRSA) 43300, *S. aureus* SC, *P. aeruginosa* 1014, *E. coli* C10E, *Enterobacter cloacae* Gd1E0, *Serratia marcescens* 5c5K. Subsequently, 10 μL of AgNPs, NaLS and AgNO_3_ were prepared in sterile water and spotted over the solid medium previously seeded with the standardized microbial suspensions. The plates were incubated at 37 °C for 24 h and then, the microbial growth inhibition diameter zone (IDZ) was measured.

#### 2.4.2 Quantitative evaluation of the antimicrobial activity of AgNPs and NaLS

The minimum inhibitory concentrations (MICs) were measured as described previously by Vlad et al. [62]. Briefly, binary serial dilutions of the stock solutions in liquid medium (Trypton Soy broth, TSB) were prepared in 96 wells plates (AgNPs/NaLS obtained from 1600 to 25 μg/mL NaLS and 274–4.28 μg/mL AgNO_3_ and 1600–25 μg/mL for NaLS). Then a volume of 10 μL of from the microbial suspension with the standard density of 0.5 Mc Farland was added to each well; positive (microbial culture) and negative (sterile culture medium) controls were used for each strain. The plates were incubated for 24 h at 37 °C. The absorbance was measured at 620 nm with FlexStation 3 UV–VIS (Molecular Devices Company, Sunnyvale, CA, USA) spectrophotometer.

#### 2.4.3 The influence of AgNPs on the microbial adherence capacity to the inert surface

The influence on the microbial adherence to the inert substratum has been tested in 96-well untreated polystyrene plates by the microtiter method. The biofilm biomass developed on the plate walls has been fixed with methanol, stained with 1% violet crystal, resuspended in acetic acid 33% and then, the absorbance of the biological material was determined at 490 nm and compared to that of the negative (sterile culture medium) and positive (untreated bacterial suspensions) controls [63]. The minimum biofilm eradication concentration (MBEC) was expressed both in relation to the concentration of NaLS and Ag^+^.

#### 2.4.4 Quantification of reactive nitrogen intermediates (RNI)

Quantification of nitrogen intermediates was determined for MIC/2 and MIC/4 by Griess reaction [64]. The bacterial suspensions, after incubation with samples 24 h at 37 °C, were centrifuged for 10 min at 10,000 rpm. To the supernatant (50 μL), 50 μL of 2% sulphanilamide in 5% (v/v) H_3_PO_4_ and 50 μL of 0.13% N-(1-naphthyl)-ethylenediamine aqueous solution were added. Azo dye was measured after 30 min at λ = 540 nm. For the quantification of nitric oxide, a calibration curve with different NaNO_2_ concentrations in the range of 5–100 μM was drawn (R^2^ = 0.9989, LOD = 0.12 μM NaNO_2_). For all sample concentrations, blanks were made consisting of medium uninoculated with sample.

#### 2.4.5 Advanced oxidation protein products (AOPP)

The concentration of microbial advanced oxidation protein products (AOPP) was evaluated by a spectrophotometric assay described by Quinteros et al. [65] with few changes. Briefly, 500 μL of bacterial suspensions cultured for 24 h in TSB at 35 °C were incubated with 500 μL of AgNP/NaLS, NaLS or phosphate saline buffer for 2 h and 4 h at 37 °C. Then, the sample taken was centrifuged at 10,000 rpm for 10 min. Over 100 μL of supernatant, 50 μL of IK (1.16 M) and 50 μL of acetic acid were added. The absorbance was read at 340 nm against a blank containing 100 μL TSB with specific concentration of samples, 50 μL KI, and 50 μL acetic acid. The AOPP content was calculated using the extinction coefficient 26 mM^−1^ cm^−1^ and expressed as μmol chloramine T equivalent/mg protein according Braik et al. [66]. The quantity of soluble protein in bacterial suspensions was determined by the Bradford assay.

### 2.5 Hemolytic index (HI)

Hemolysis assay was performed on sheep red blood cells (RBC). In this purpose, 9 mL of the blood sample and 1 mL of 10% citric acid dextrose were mixed to prevent blood clotting. The tube was then centrifuged at 3000 rpm for 5 min. The supernatant containing platelet-poor plasma was discarded and the pellet containing RBC was resuspended in 10 mL of phosphate buffer saline (PBS, 0.1 M, pH 7.4). The process was repeated three times. Finnally, the cells were suspended in PBS. The applied method has been described by Das et al. [25] with slight modifications. Thus 500 μL of different concentrations of NaLS and AgNP/NaLS (adjusted to 0.9% NaCl) were mixed with 250 μL of erythrocytes suspension. The tubes were then gently inverted and incubated at 37 °C for 60 min. Positive and negative controls were prepared by adding the same amount of erythrocyte suspension to deionized water and PBS, respectively. After incubation, the samples were centrifuged at 3000 rpm for 5 min and the supernatant was then carefully distributed in 96-well plates. The absorbance at 540 nm of the supernatant was measured with FlexStation 3 UV–VIS (Molecular Devices Company, Sunnyvale, CA, USA) spectrophotometer. The percentage of hemolytic index (HI%) was calculated according to:

(1) Hemolysis(%)=(Asample−Acontrol(−))/(Acontrol(+)−Acontrol(−))x100

### 2.6 Antioxidant activity

#### 2.6.1 DPPH assay was performed according to the method described by G. Madhu et al. [67] with slight changes

The reaction mixture consisted of adding 50 μL of sample/standard and 50 μL of 0.3 mM DPPH radical methanolic solution. The absorbance was measured at λ = 517 nm after 20 min of incubation in the dark and 5 min centrifugation at 7000 rpm, using a UV–VIS spectrophotometer, Mulsiskan FC instrument (Thermo Scientific). The concentrations used for the Trolox calibration curve were in the range of 5–80 μM (R^2^ = 0.9975).

#### 2.6.2 The FRAP assay was performed by the method described by Thaipong et al. [68]

The reaction mixture consisted of adding 25 μL of sample/standard and 475 μL of FRAP regent (prepared according to Multescu et al. [69]). The absorbance was measured at λ = 593 nm after 20 min of incubation in the dark at 37 °C and 5 min centrifugation at 7000 rpm, using a UV–VIS spectrophotometer (Mulsiskan FC instrument (Thermo Scientific)). A 1 mM Trolox stock solution was used to plot the calibration curve, the concentration ranged between 30 and 250 μM Trolox (R^2^ = 0.9978).

#### 2.6.3 The *CUPRAC assay* was performed according to a method described by celik et al. [70]

60 μL of sample/standard solutions of different concentrations were mixed with 50 μL CuCl_2_ (10 mM), 50 μL neocuproin (7.5 mM), and 50 μL ammonium acetate buffer 1 M, pH = 7.00. After 30 min, the absorbance was measured at 450 nm. The concentrations used for the Trolox calibration curve were in the range of 0.125–1.5 mM (R^2^ = 0.9983).

### 2.7 Biocompatibility/cytotoxicity

The biocompatibility/cytotoxicity of AgNP/NaLS was tested on human keratinocyte line, HaCaT cells and cancer non-melanoma cell line, Cal-27. The both cell lines were cultured in DMEM medium (Dulbecco's Modified Eagle Medium, Sigma-Aldrich) supplemented with 10% fetal bovine serum (Sigma-Aldrich) and 1% Pen/Strep (penicillin/streptomycin solution, 50 μg/mL – Sigma – Aldrich) for 24 h at 37 °C, 95% humidity with 5% CO_2_. Cells were washed with phosphate buffered saline (PBS, Sigma Aldrich), trypsinized (0.25% trypsin- 0.53 Mm EDTA, Thermo Scientific) and counted using Trypan Blue and a Burker-Turk counting chamber. The nanoparticles were co-cultured with the cells (previously seeded at a density of 5 × 10^5^ cells/well) for 24 h (37 °C, 95% humidity, 5% CO_2_).

#### 2.7.1 The MTT assay was used to assess cell viability and proliferation in the presence of nanoparticles

This viability test allows the quantitative evaluation of live cells in culture. The compound MTT [3-(4,5-dimethylthiazol-2-yl)-2,5-diphenyltetrazolium] is permeable to living cell membranes and metabolized into soluble formazan crystals. The cells were incubated at 37 °C, 95% humidity with 5% CO_2_. After incubation, formazan crystals were solubilized with SDS-HCl buffer and absorbance was read at 550 nm on a Flex Station 3 instrument.

#### 2.7.2 Lactate dehydrogenase (LDH) release assay

The culture medium was collected after 24 h of incubation with the NPs, and LDH release was measured using Cytotoxicity Detection Kit PLUS (Roche, USA) according to manufacturer's instructions. Volumes of 50 μL culture supernatants were mixed with 50 μL reaction mixture of catalyst, dye solution, and incubated for 30 min in a dark place. The reaction was stopped with 50 μL of stop solution and the absorbance was read at 490 nm using a microplate reader (Flex Station 3, Molecular Devices, USA).

#### 2.7.3 Live-Dead test was carried out as an alternative method to evaluate the cytotoxicity of different types of NPs

The Live-Dead test (Thermo Scientific) is a qualitative test that contains calcein - AM (a green, fluorescent compound, permeable only to living cells, labeling the live cells) and ethidium homodimer-1 (an intercalating agent that stains dead cells). Evaluation of Cal-27 cells was performed on an Olympus IX73 fluorescence microscope.

### 2.8 Statistical analysis

Data were expressed as means ± SD determined by triplicate analysis. The statistical analysis was conducted using GraphPad Prism v9. Data were analyzed using ordinary two-way ANOVA and Sidak's multiple comparisons test with individual variances computed for comparison between samples (AgNP/NaLS, AgNO_3_, NaLS) and positive control (microbial strain) for qualitative antimicrobial and antioxidant activities, NO release, AOPP and hemocompatibility. The biocompatibility and cytotoxicity assays were analyzed by ordinary two-way Anova using a two-stage linear step-up procedure of Benjamini, Krieger, and Yekutieli, with a single pooled variance method. The level of statistical significance was set at *P* < 0.05.

## 3 Results

Several arrays of experimental data have been obtained to argue the potential use of AgNPs/NaLS systems as readily available bioactive agents in wound management. All physico-chemical analyses were carried out on pristine or diluted raw solutions, without any purification step by centrifugation, washing, filtration, dialysis or other mean to emphasis the feasibility of this synthesis method in obtaining a useful nanoparticulate system. Information regarding the formation and stability of these systems are also included from applicative perspective.

### 3.1 The role of NaLS and synthetic conditions on AgNPs formation

Due to the yellow brownish color of the NaLS solutions before the AgNO_3_ addition, the on-going reaction is relatively difficult to visually follow [71]. However, due to the specific surface plasmon resonance (SPR) band exhibited by AgNPs, the UV-VIS spectroscopy is a very convenient way to monitor the formation and stability of silver colloids as well as the influence of reaction parameters [72]. It is generally accepted that the absorption maxima of relatively spherical, non-aggregated AgNPs with sizes up to 50–60 nm are mainly located in the range of 400–440 nm [24,73,74], but SPR intensities and maximum wavelengths might vary as result of the complex interplay between nanoparticles characteristics like size, shape, concentration, chemical surrounding, and polydispersity [75,76] https://doi.org/10.1002/ppsc.201400117. Despite this variability, the UV-VIS spectroscopy could still give a correct, simple, and fast approximation of the reaction output [77].

**Fig. 1:**
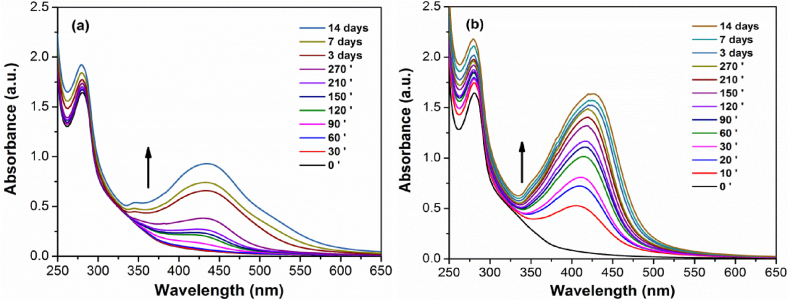
Time evolution of SPR band for AgNPs/NaLS solutions during synthesis and later storage at 25 °C for reactions made at: a) 25 °C, 24 h; b) 70 °C, 2 h.

When AgNO_3_ and NaLS solutions are mixed at 25 °C, the SPR band occurs in about 90 min (Fig. 1a). Its intensity increased in time but failed to reach a maximum even after two weeks at room temperature with intermittent shaking. Also, the absorbance increase is accompanied in the first hours with a weak redshift in the maximum wavelength (*λ*_max_) from around 420 nm up to about 435 nm (see Supplementary Material, Fig. S1). The *λ*_max_ values remain almost constant later, whereas the absorbance bandwidth broadens, and shoulders begin to appear after 3–7 days (Fig. 1a).

These observations demonstrate the successful formation of AgNPs with size of tens of nanometers, but also pointed out to a relative high polydispersity and agglomeration after long storage [78] apparently due to the impossibility of NaLS to stop the nanoparticle ripening and aggregation. In fact, polydispersity should originate in part in the slow nucleation stage, which adds lag times in nanoparticle growth. It thus became a limiting factor, favoring the simultaneous presence of a large dimensional range of colloids and subsequent non-uniform, stepped maturation.

The strong influence of the nucleation rate on AgNPs formation was confirmed by the absorbance evolution dynamics observed when syntheses were carried out at 70 °C for 2 h, and the reaction mixtures were afterwards maintained at room temperature with intermittent shaking (Fig. 1b). After only 10 min of reaction at 70 °C the absorbance values exceed those observed after 24 h at 25 °C, and after 1 h those measured after 2 weeks at 25 °C. The wavelengths corresponding to the absorbance maxima are 10–20 nm lower when reaction was carried out at higher temperature but are also redshifted in time from around 400 nm to about 425 nm (see Supplementary Material, Fig. S2). This indicates that a higher reaction temperature allows the formation of smaller nanoparticles but does not halt completely their increase in size over time. However, the absorbance tail through higher wavelengths that slowly forms during storage is virtually absent after two weeks of storage in the case of reaction occurred at 70 °C, which suggests a better stability of AgNPs against aggregation.

It must be mentioned here that in the case of AgNPs the absorbance increase is not directly proportional with the formation of new nanoparticles due to the positive contribution of the nanosphere albedo, which represents the ratio of light scattering to total extinction [79]. The albedo increases non-linearly by an order of magnitude for a rise in nanoparticle diameter from 10 to 100 nm. Although the enhancement in absorbance due to increased size of nanoparticle is much lower than the effect given by new particle formation, the albedo still has non-negligible effects on the absorbance readings. These effects could be observed in Fig. 1, where the absorbance maximum still slightly increases after several days of storage at 25 °C.

**Fig. 2:**
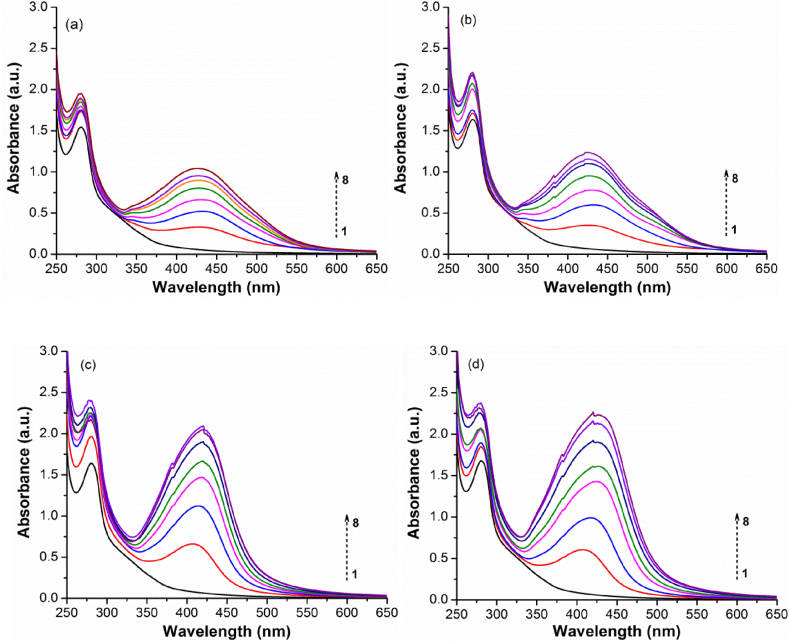
Influence of AgNO_3_ concentrations on AgNPs synthesis. UV–Vis spectra recorded after a) 1 day, and (b) 7 days at 25 °C; (c) 2 h at 70 °C, and (d) 2 h at 70 °C followed by 7 days at 25 °C (10 fold dilution).

**Fig. 3:**
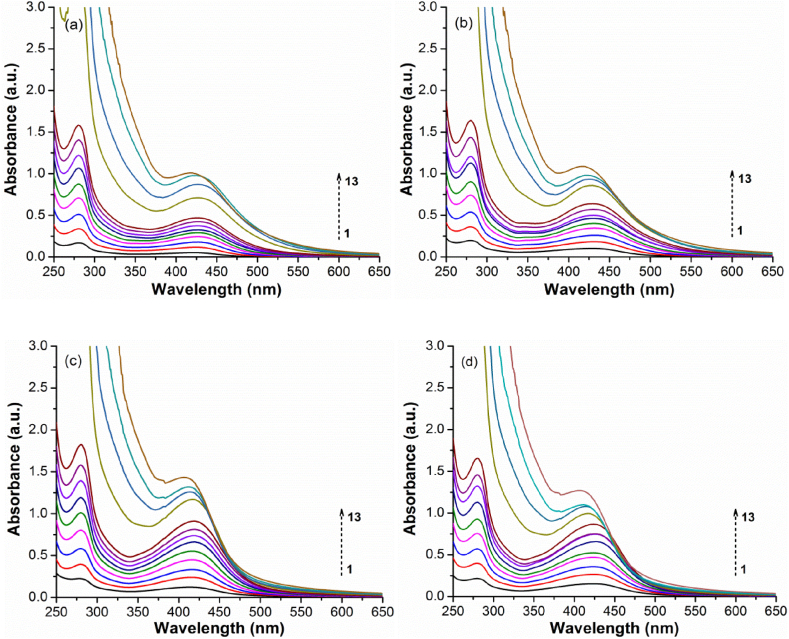
Influence of NaLS concentration on AgNPs synthesis. UV–Vis spectra recorded after a) 1 day, and (b) 7 days at 25 °C; (c) 2 h at 70 °C, and (d) 2 h at 70 °C followed by 7 days at 25 °C (20 fold dilution).

The combined effects of phenolic group deprotonation, quinone formation, and new cation-carbonyl/cation-π interactions that occur during AgNPs nucleation and growth are substantially masked in the UV–Vis spectra due to the strong signal given by nanoparticle formation. However, these processes conduct to enhancements in the π-π* and n-π* transitions related to the lignosulfonate aromatic groups that are reflected in their spectra (Fig. 1) through a stronger absorbance at 280 nm, a vague shoulder at around 350 nm, and by a thin absorbance tail that smoothly starts to lengthen up to around 600 nm simultaneously with the onset of AgNPs formation [[80], [81], [82]]. Since AgNPs formation is accompanied by significant proton release, it lowers the pH from 8.7 ± 0.1 to 4.5 ± 0.2 for both reaction temperatures, resulting in an acidic character of AgNPs/NaLS colloidal solutions. A survey of AgNO_3_ and NaLS concentrations (Table 1; Fig. 2a–d, Fig. 3a–d) suggests that each mg of NaLS could efficiently reduce in these experimental conditions up to at least 5 mmol of silver ions with subsequent formation of AgNPs. At higher ratios, the process tends to take longer times, to become incomplete, non-uniform, and to promote the nanoparticle agglomeration.

On the other hand, the increase in polymer concentrations is permissive up to at least 10–12 mg/mL but could not be raised further due to the strong increase in the NaLS tendency to self-aggregate. The obtained results prove that the simple and green synthesis method proposed herein is indeed an effective one, and infirm previous assumptions [46,47] considering that strong alkaline media are required for such kind of reactions.

### 3.2 AgNPs/NaLS structural characteristics

**Fig. 4:**
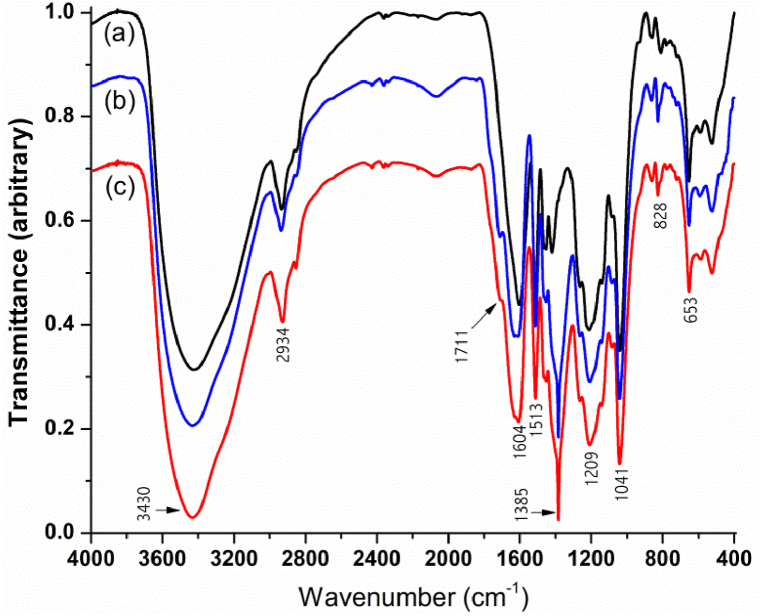
ATR FT-IR spectra of NaLS (a) and solid AgNPs/NaLS mixtures obtained after synthesis at 70 °C (b) and room temperature (c).

AgNPs formation is evidenced by the very strong band centered at 1385 cm^−1^ (Fig. 4b and c), which is characteristic for the free NO_3_^−^ ions separated of their heavy metallic counterparts, as opposed to the lower wavelength of 1376 cm^−1^ attributed to the Ag ^+^ NO_3_^−^ ion pair [83]. On the other hand, a partial oxidation of lignosulfonate phenol groups to quinone is suggested by the slight narrowing and reduction of O–H stretching broad band from around 3430 cm^−1^, combined with the broadening of aromatic skeletal vibrations band from 1604 cm^−1^ through higher wavelengths and rising of a small shoulder at 1711 cm^−1^, both attributable to the stretching of newly formed carbonyls (Fig. 4a–c).

The absorbances related to the lignosulfonate main aromatic structural units, syringyl (3,5-dimethoxy-4-hydroxyphenylpropane) and guaiacyl (4-hydroxy-3-methoxyphenylpropane), are all slightly reduced and shifted with 1–3 cm^−1^ due to the presence of AgNPs. As expected, the signals given by less substituted guaiacyl rings (1513 cm^−1^, aromatic skeletal vibrations; 861/810 cm^−1^, C–H out-of-plane deformation) are more affected than those corresponding to syringyl (1604 cm^−1^, aromatic skeletal vibrations; 828 cm^−1^, C–H out-of-plane deformation). In addition, the band centered at 1212 cm^−1^ mainly given by C–O stretching vibrations is blue shifted to 1209 cm^−1^ due to their partial involvement in AgNPs coordination.

No significant differences could be observed in the spectra of AgNPs/NaLS mixtures obtained at 25 °C and 70 °C, suggesting that temperature affects only the speed of the silver ion reduction and the processes related to the nanoparticle growth. Likewise, the pattern of symmetrical stretching assigned to methylene groups from about 2934 cm^−1^ [84] is virtually unchanged, which means that phenylpropanoid backbone is not affected by AgNPs/NaLS system formation.

The distinct peaks determined by stretching vibrations associated with sulfonic functional groups (S=O, 1041 cm^−1^; S–O, 653 cm^−1^) [85,86] are not shifted at all, suggesting that while they are somewhat susceptible for oxidation during silver ion reduction, do not directly participate in the later capping of nanoparticles. The sulfonic functional groups and their spatial displacement have instead a very important role in the solution stabilization of both pure and AgNPs-embedded NaLS through the electrostatic repulsion between negatively charged micelles and nanoparticles.

### 3.3 Morphology and solution stability

The potential of NaLS to form colloidal micelles was tested before analyzing the zeta potential and size distribution of AgNPs/NaLS assemblies. Notable differences were observed between the behavior of NaLS in concentrated and diluted aqueous solutions (see Supplementary Material, Fig. S3). While in the case of stock solutions of 2 mg/mL, NaLS tends to form colloids with dimensions in the range of 0.3–1.0 μm and narrow zeta potential values around −32.6 mV, at 20 mg/mL it forms colloids within a dimensional range of 0.9–1.7 μm and shows broad zeta potential values centered at about −23.4 mV. From a theoretical point of view suspensions are considered stable when the zeta potential absolute value exceeds 15–30 mV, depending on the type of the colloids [73]. Thus, NaLS shows a relative stability in diluted aqueous solutions, which further declines as concentration increases.

**Fig. 5:**
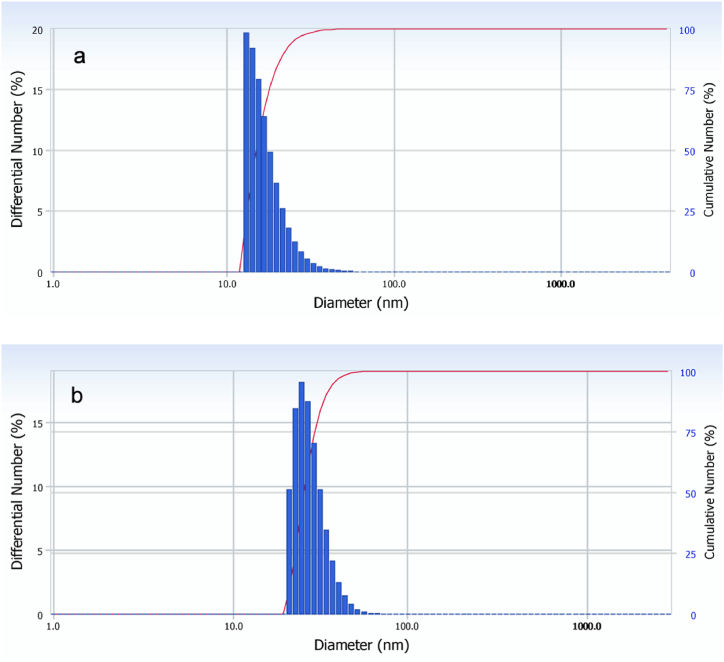
Size number distribution of AgNPs/NaLS colloidal systems obtained at 70 °C after one week (a), respectively after 15 months (b).

In the case of AgNPs/NaLS solutions the zeta potentials were found to depend in a small extent on the preparation temperature (−27.1 mV for 25 °C; −27.5 mV for 70 °C). The average size of colloidal nanoparticles acquired from DLS measurements (made at one week after the onset of syntheses) almost halved, from more than 30 nm to less than 20 nm, while the polydispersity index increases when temperature of reaction is switched from 25 °C to 70 °C. The high polydispersity is mainly due to the absence of a preliminary purification step, to the previously discussed lignosulfonate behavior during and after silver ions reduction and to the raw polydispersity of its three-dimensional polymer chains. The moderate stability of AgNPs/NaLS solutions at storage as liquid dispersions at room temperature may also add its own contribution to these results. While the low reaction rate and stability makes the 25 °C synthesis questionable from applicative point of view, results obtained for the 70 °C process are noteworthy. Thus, the average of initial hydrodynamic size number distribution is of about 18 ± 5.1 nm and rise to only 28.6 ± 6.0 nm after 15 months of storage at dark and 4 °C (Fig. 5). Moreover, the zeta potential is even lower (−32.9 mV) for older AgNPs/NaLS and comparable with the value obtained for diluted, fresh NaLS solutions, which hints to a better exposure of sulfonic moieties, an increase in the amount of free acidic groups, and perhaps some limited macromolecule degradative oxidation associated with carboxyl formation. Nevertheless, while the nanoparticulate solutions are clear and almost free of deposits after more than one year, the pure NaLS solutions within the same concentration range decay and became turbid after only a couple of weeks. This fact suggests the occurrence of a reciprocal stabilization between the system components.

**Fig. 6:**
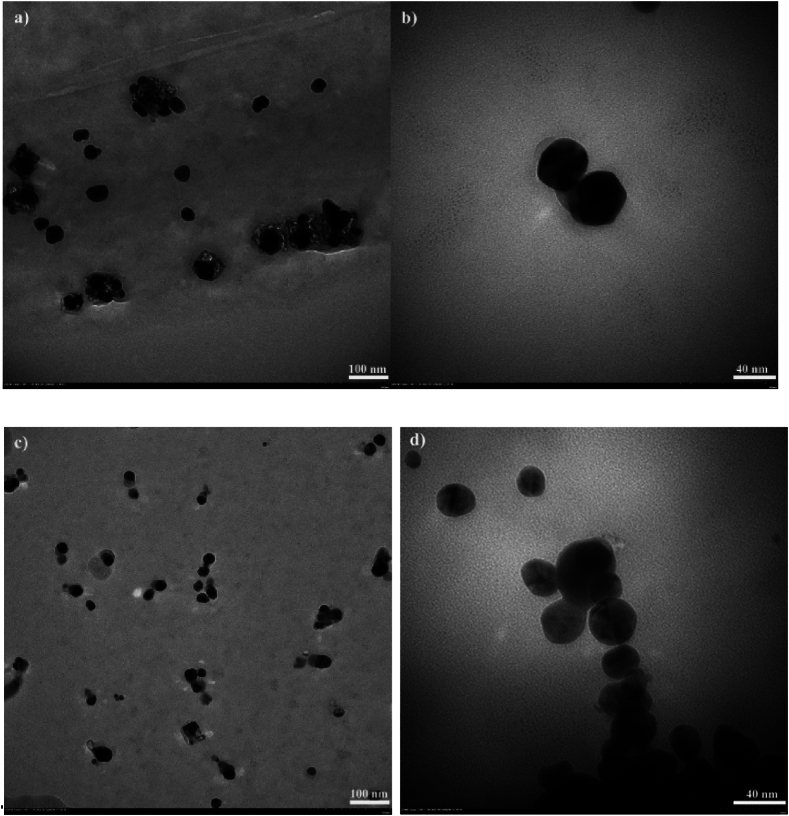
TEM images of AgNPs formed in the presence of NaLS at 25 °C (a, b); 70 °C (c, d).

**Fig. 7:**
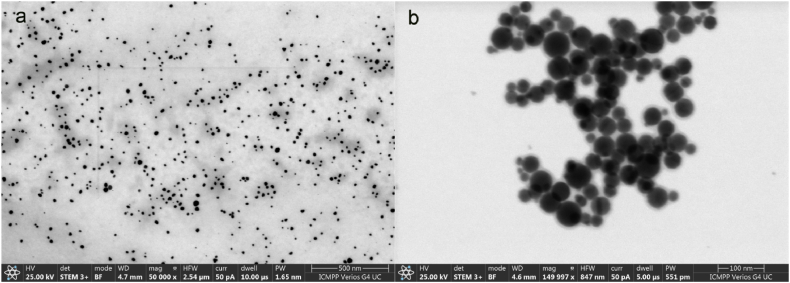
AgNPs morphology as seen in FESEM images obtained on 500 nm (a) and 100 nm (b) scale (70 °C synthesis).

The particle size distributions and polydispersity are in excellent agreement with UV–Vis data and TEM (Fig. 6a–d), respectively FESEM images (Fig. 7a and b). Thus, as shown in the TEM images made one week after the onset of synthesis, the size of AgNPs tend to become smaller, while polydispersity is higher at 70 °C. TEM images (Fig. 6b–d) also suggest that the relative spherical particles have in most cases bipyramid geometries with hexagonal bases and sizes generally ranging from about 10 nm and up to 30–40 nm.

**Fig. 8:**
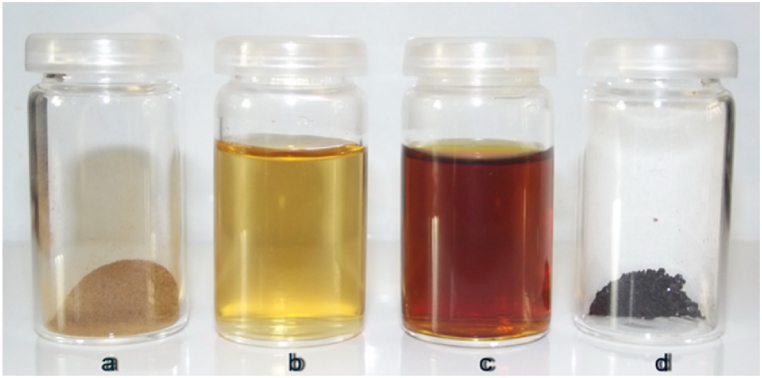
Visual presentation of NaLS solid (a) and diluted (b), and resulting AgNPs/NaLS dispersions (c) and solid form (d).

A visual comparison between raw NaLS (solid and water-diluted) and AgNPs/NaLS resulted after synthesis (dispersion and desiccated solid) is given in Fig. 8a–d. Besides the color change induced by AgNPs formation, it is interesting to note the transformation of sticky greyish dust (NaLS) into a non-stick black granular material (AgNPs/NaLS) with glassy metallic appearance.

### 3.4 Crystallinity and elemental composition of AgNPs/NaLS

**Fig. 9:**
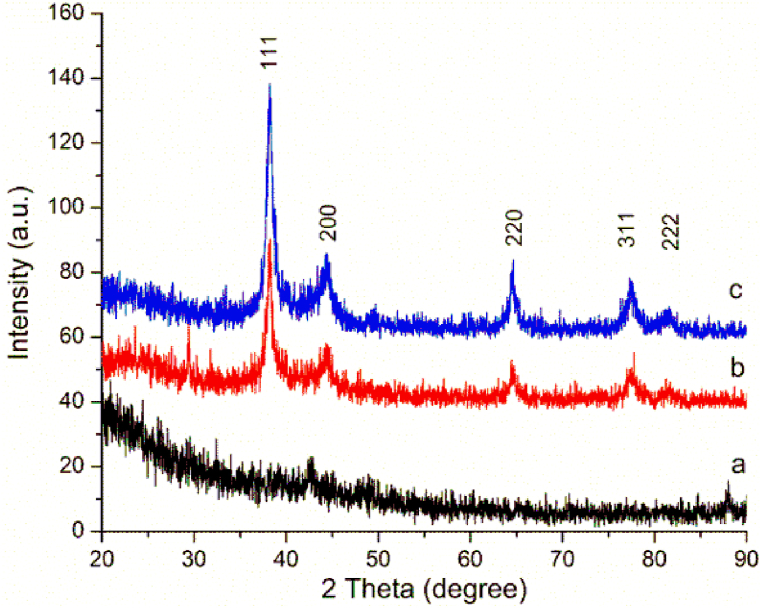
XRD patterns of NaLS (a) and AgNPs/NaLS synthesized at 25 °C (b), and 70 °C (c).

The formation of silver crystallites is clearly confirmed by the analysis of XRD data (Fig. 9b and c). The Braggs reflections seen at 2θ values of 38.170, 44.420, 64.580, 77.430, and 81.490 correspond to the well-known (111), (200), (220), (311), and (222) lattice planes of face-centered cubic silver nanocrystals (Joint Committee on Powder Diffraction Standards File no. 04–783) [71,87,88]. The presence of an intense noise in all XRD patterns (Fig. 9a–c) could be the result of various nano-crystalline domains generated by the self-assembly of polyphenolic polymer [89], relatively low silver content, and eventual traces of salts like Na_2_SO_4_ from the NaLS raw material. Nevertheless, quantifiable diffraction peaks for silver oxide or other metallic salts could not be evidenced. All diffraction peaks are broad due to the presence of small, nano-sized particles [90].

For a better overview of the nanoparticles structure and their interactions established with the capping polymer, NaLS and AgNPs/NaLS were furthermore examined by X-ray photoelectron spectroscopy (XPS). The survey scan spectra are shown in the supplementary material (see Supplementary Material, Figs. S4–S6).

**Fig. 10:**
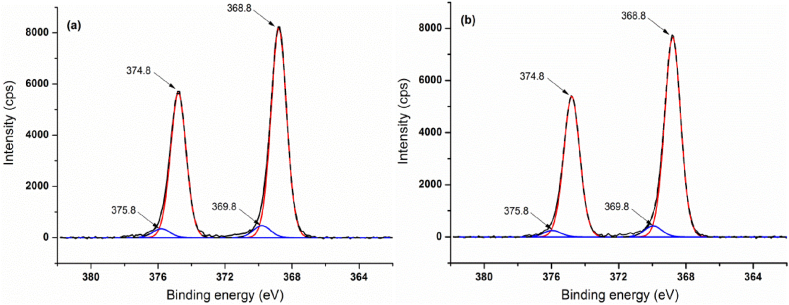
XPS spectra of Ag 3d for AgNPs/NaLS synthesized at 25 °C (a), and 70 °C (b).

The high-resolution spectra of Ag 3d5/2 and Ag 3d3/2 unveil two strong peaks at 368.8 and 374.8 eV responsible for up to 95% from the entire silver concentration and two satellite peaks at 369.8 and 375.8 eV, respectively (Fig. 10a and b). These peaks are highly blue shifted as compared with those of the bulk silver (367.9 and 373.9 eV), but each pair is split by 6.0 eV, which is an indicative of metallic silver [91]. Binding energies of 368.8 eV were previously reported for silver charge transfer states [92] and silver nanoparticles [93]. The even higher binding energies shown by the satellites could be attributed to the presence of a nanoparticle fraction with very small, sub-nanometer sized domains that may favor optical transitions conducting to luminescence and enhanced Raman scattering [94]. Moreover, these nanoparticles have a significant contribution to the high polydispersity evidenced by DLS measurements.

It is important to note that the XPS high resolution spectra of both silver and oxygen (see Supplementary Material, Fig. S7) lack signals attributable to silver oxides, in perfect agreement with the XRD data. The comparison of C 1s (see Supplementary Material, Fig. S8), O 1s, and S 2p spectra (see Supplementary Material, Fig. S9) recorded for NaLS and AgNPs/NaLS shows on the other hand, a general blue-shift of binding energies with about 0.4–0.7 eV in the presence of nanoparticles, confirming their interactions with the capping material. In addition, synthesis at different temperatures does not add significant discrepancies other than those that could be caused by NaLS behavior at evaporation.

### 3.5 Antimicrobial activity

Reference and resistant strains belonging to *S. aureus,* enterococci, *P. aeruginosa* and Enterobacterales were selected for assessing the antibacterial potential of the obtained nanoparticles.

The qualitative screening of AgNP/NaLS revealed their similar activity against all Gram-positive and Gram-negative tested strains, diameters of the growth inhibition zone (DZI) presented in Table 2 ranging from 8.67 ± 0.52 to 10.67 ± 0.52 mm. The NaLS did not induce any inhibition of bacterial growth on solid media; therefore, it did not show any antibacterial activity in this assay. The antibacterial activity of AgNP/NaLS has been higher than that of AgNO_3_ in the case of 8 of the 10 tested strains, the differences being statistically significant for the following strains: MRSA 43300 (*P* < 0.0001), *P. aeruginosa* ATCC 27853 (*P* < 0.05), *S. marcescens* 5c5K (*P* < 0.05) and *E. coli* C10E (*P* < 0.05). The results of the agar diffusion assay thus suggest the synergic antibacterial effects of the AgNP and NaLS components.

**Table 2:** The DZI (mm) values of the AgNP/NaLS, NaLS and AgNO_3_.

No.	Strains	DZI (mm)			
		AgNP/NaLS	NaLS	AgNO_3_	P-Value
1.	*S. aureus* ATCC 25923	9.50 ± 1.05	NA^a^	9.75 ± 0.96	>0.05
2.	*E. faecalis* ATCC 29212	9.50 ± 1.05	NA^a^	9.17 ± 0.98	>0.05
3.	*S. aureus* SC	9.67 ± 0.52	NA^a^	9.25 ± 1.26	>0.05
4.	*MRSA* 43300	9.17 ± 0.98	NA^a^	5.00 ± 0.82	<0.0001
5.	*P. aeruginosa* ATCC 27853	8.67 ± 0.52	NA^a^	5.75 ± 0.96	<0.05
6.	*E. coli* ATCC 25922	9.67 ± 0.12	NA^a^	9.75 ± 0.50	>0.05
7.	*P. aeruginosa* 1014	9.50 ± 1.38	NA^a^	7.75 ± 0.5	>0.05
8.	*S. marcescens* 5c5K	10.17 ± 0.98	NA^a^	8.25 ± 0.50	<0.05
**9.**	*E. cloacae* G d1E0	10.50 ± 0.55	NA^a^	8.75 ± 0.50	>0.05
**10**	*E. coli* C10E	10.67 ± 0.52	NA^a^	8.75 ± 0.96	<0.05

a NA-Not active.

In the liquid medium quantitative assay allowing the assessment of MIC values, AgNP/NaLS have also exhibited a better activity than NaLS. The MIC of obtained AgNP/NaLS varied between 800 and 50 μg/mL, considering the NaLS concentration or 137–8.56 μg/mL considering the Ag^+^ concentration in the diluted suspensions corresponding to the MIC values, with lower values recorded on the Gram-negative bacterial strains: *P. aeruginosa* ATCC 27853, *E. coli* C10E, *E. coli* ATCC 25922, *S. marcescens* 5c5K and *E. cloacae* Gd1E0 (Table 3).

**Table 3:** Minimum inhibitory concentration (MIC), Minimum bactericidal concentration (MBC) and Microbial biofilm eradication concentration (MBEC) of AgNP/NaLS expressed as Ag^+^ and NaLS concentrations at the respective dilutions.

Strains	Expressed as concentration of	MIC (μg/mL)	MBC (μg/mL)	MBEC (μg/mL)
***S. aureus* ATCC 25923**	Ag^+^	137	137	137
	NaLS	800	800	800
***E. faecalis ATCC 29212***	Ag^+^	137	>137	137
	NaLS	800	>800	800
***S. aureus* SC**	Ag^+^	137	>137	68.5
	NaLS	800	>800	400
***MRSA* 43300**	Ag^+^	137	137	68.5
	NaLS	800	800	400
***P. aeruginosa ATCC* 27853**	Ag^+^	8.56	17.125	8.56
	NaLS	50	100	50
***E. coli ATCC* 25922**	Ag^+^	17.125	17.125	17.125
	NaLS	100	100	100
***P. aeruginosa* 1014**	Ag^+^	137	137	137
	NaLS	800	800	800
***S. marcescens* 5c5K**	Ag^+^	34.25	137	68.5
	NaLS	200	800	400
***E. cloacae* G d1E0**	Ag^+^	34.25	137	17.125
	NaLS	200	800	100
***E. coli* C10E**	Ag^+^	34.25	68.5	17.125
	NaLS	200	400	100

**Fig. 11:**
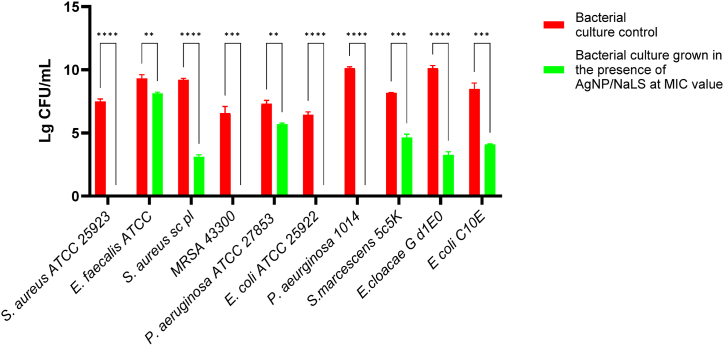
Representation of the intensity of the antibacterial effect of AgNPs related to the density of the initial bacterial suspension.

To confirm whether the MIC values coincide with MBC, the viable cells count (CFU/mL) were evaluated by spotting ten-fold dilution of the well corresponding to the MIC values and previous ones on agar medium and incubation for 24 h at 37 °C. For the *S. aureus* ATCC 25923, MRSA 43300, *P. aeruginosa* 1014 and *E. coli* ATCC 25922 strains, the MIC values coincided with the MBC ones for AgNP/NaLS, while for *S. aureus* SC (*P* < 0.0001), *P. aeruginosa* ATCC 27853 (*P* < 0.01), *S. marcescens* 5C5K (*P* < 0.001), *E. cloacae* Gd1E0 (*P* < 0.0001) and *E. coli* C10E (*P* < 0.001) were significantly lower (Fig. 11). The MBC values ranged between 800 and 100 μg/mL for AgNP/NaLS expressed as NaLS and 137–17.125 μg/mL expressed as Ag^+^ concentration (Table 3). When comparing the lg CFU/mL of the initial suspension for each microbial strain with the lg CFU/mL obtained in the presence of the MIC value of the tested nanoparticles, a decrease of 2 up to 10 lg has been observed, demonstrating their excellent antibacterial activity.

To highlight the antibiofilm effect, the microbial adhesion to the inert substrate represented by the polypropylene microwells was evaluated. Thus, inhibition of adhesion was considered only if the MBEC value was lower than the MIC value and implicitly the MBC (Table 3).

**Fig. 12:**
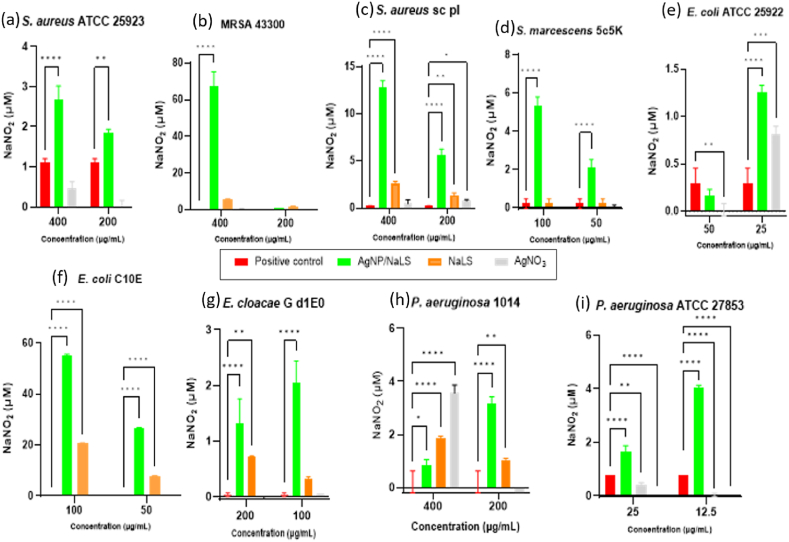
Reactive nitrogen intermediates release determined by Griess's reaction for bacterial strains treated with AgNPs/NaLS concentrations at MIC/2 and MIC/4. The bacterial strains used for this assay were both reference (*S. aureus* ATCC25923 (a), MRSA 43300(b), *E. coli* ATCC 25922 (e), *P. aeruginosa* ATCC 27853 (i)) and isolated from the clinic (*S. aureus* sc pl (c), *S. marcescens* 5c5k (d), *E. coli C10E* (f), *E. cloacae* G d1E0 (g) and *P. aeruginosa* 1014 (h)). All variants in the case of the *E. faecalis* ATCC 29212 strain had values below the detection limit and are not represented. The individual components ratio of AgNP/NaLS is 5.84:1 for NaLS: AgNO_3_, thus 1 mL of AgNP/NaLS results from 1.6 mg NaLS and 274 μg AgNO_3_. The concentrations are expressed according to the NaLS content.

Taking into account that one of the mechanisms by which AgNPs could inhibit the microbial adherence is represented by the increase of NO release in the extracellular environment, we have investigated the effect of sub-inhibitory concentrations (MIC/2 and MIC/4) of AgNP/NaLS on the release of reactive nitrogen species in all tested strains (Fig. 12a–i). In our study, both tested concentrations of NaLS increased the release of reactive nitrogen species in case of *S. aureus* SC, *E. coli* C10E, *E. cloacae* Gd1E0 and *P. aeruginosa* 1014 strains. The MIC/2 of AgNO_3_ stimulated the production of nitrogen reactive species in the case of *P. aeruginosa* 1014, while the MIC/4 of AgNO_3_ in the case of *E. coli* ATCC 25922.

**Fig. 13:**
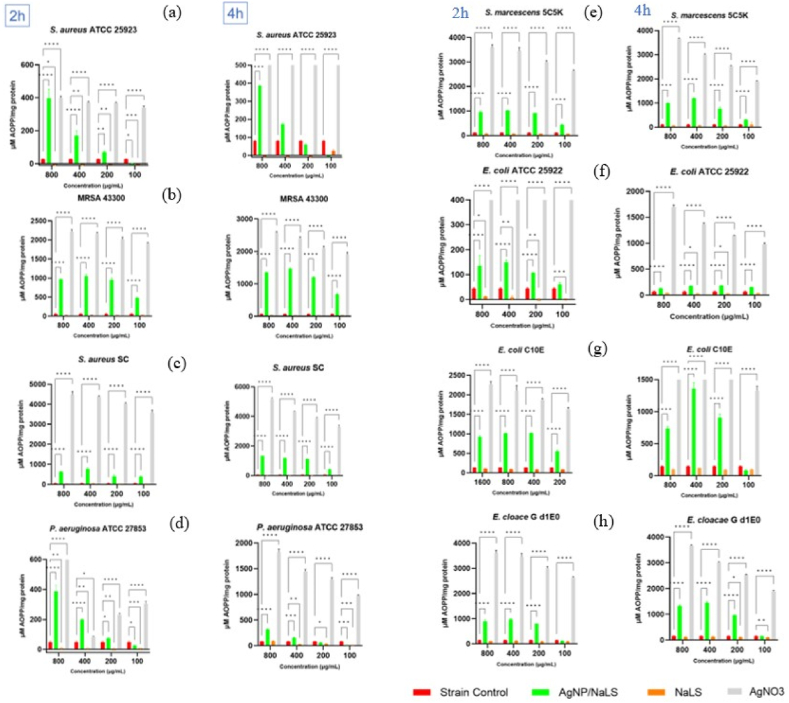
Oxidation Protein Products (AOPP) in bacterial cells treated with AgNP/NaLS, NaLS and AgNO_3_ compared to the control strains for 2 h (left) and 4 h (right). The microbial strains used were *S. aureus* ATCC 25923 (a), MRSA 43300 (b), *S. aureus* SC (c), *P. aeruginosa* ATCC 27853 (d), *S. marcescens* 5C5K (e), *E. coli* ATCC 25922 (f), *E. coli* C10E (g) and *E. cloacae* G d1E0 (h). The individual components ratio of AgNP/NaLS is 5.84:1 for NaLS: AgNO_3_, thus 1 mL of AgNP/NaLS results from 1.6 mg NaLS and 274 μg AgNO_3_. The concentrations are expressed according to the NaLS content (*P < 0.05, **P < 0.01, ***P < 0.001, ****P < 0.0001).

For all strains, the AOPP levels, at two different times (2 h and 4 h), increased proportionally with the AgNO_3_ concentration (*P* < 0.0001), confirming that AgNO_3_ could act as an exogenous NO donor (Fig. 13a–h). These results are similar with those reported in another study [95]. However, the presence of NaLS in the composition of AgNP/NaLS induces a drastic decrease of AOPP levels indicating a decreased protein oxidation, as compared to AgNO_3_, especially in the case of (*S. aureus* ATCC 25923 (*P* < 0.05), *P. aeruginosa* ATCC 27853 (*P* < 0.01), *S. marcescens* 5C5K (*P* < 0.05), *E. coli* ATCC 25922 (*P* < 0.05, *P* < 0.01, *P* < 0.001)).

### 3.6 Hemocompatibility

The AgNPs/NaLS showed overall less hemolytic activity (<5%) than NaLS and the hemolysis index increased with concentration. Since a maximum of 5% hemolysis is allowed for biomaterials, the AgNP/NaLS nanoparticles are safe up to a concentration of 1.6 mg/mL expressed according to the NaLS content, proving their biocompatibility within the assessed bioactive concentration rang.

**Fig. 14:**
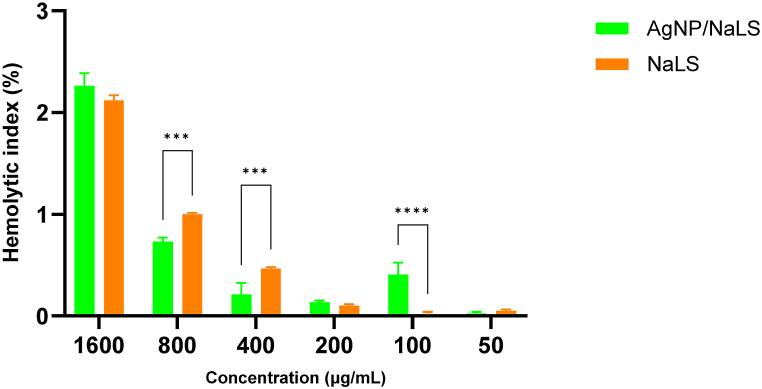
Hemolysis of erythrocyte suspension by AgNP/NaLS and NaLS. Results are in form: mean ± standard deviation, each experiment was performed in triplicate. The ordinary two-way ANOVA – Sidak's multiple comparisons test, revealed significant differences between AgNP/NaLS vs NaLS (*P < 0.05, **P < 0.01, ***P < 0.001, ****P < 0.0001). The individual components ratio of AgNP/NaLS is 5.84:1 for NaLS: AgNO_3_, thus 1 mL of AgNP/NaLS results from 1.6 mg NaLS and 274 μg AgNO_3_. The concentrations are expressed according to the NaLS content.

From Fig. 14 it can be seen that NaLS has a significantly higher hemolytic index compared to AgNP/NaLS for concentrations of 800 μg/mL (*P* < 0.01) and respectively 400 μg/mL (*P* < 0.05) but the values are still significantly below the threshold of 5%. At the highest concentration (1600 μg/mL), a higher hemolysis can be distinguished for AgNP/NaLS, but without statistical significance.

### 3.7 Antioxidant activity

**Fig. 15:**
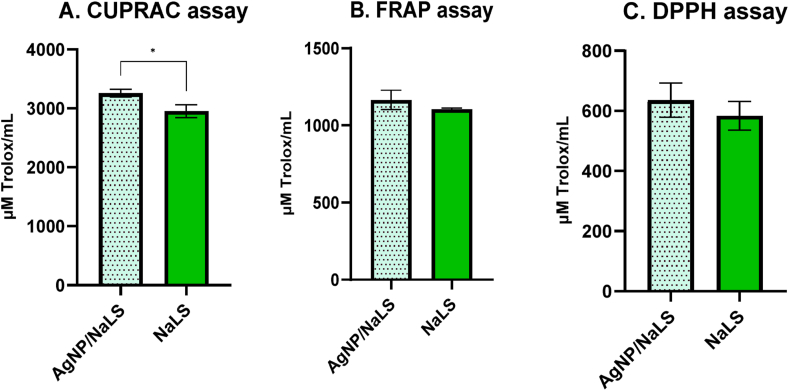
Comparative antioxidant activity between NaLS and AgNP/NaLS by CUPRAC (A), FRAP (B) and DPPH (C) assays; 1 mL of AgNP/NaLS results from 1.6 mg NaLS and 274 μg AgNO_3_.

The antioxidant activity was determined for AgNP/NaLS and NaLS (at a concentration corresponding to that found in AgNP/NaLS) by CUPRAC, FRAP and DPPH methods. A similar antioxidant activity of NaLS and AgNP/NaLS was obtained in the DPPH (using an organic radical) and FRAP (using an acidic medium) assays (Fig. 15), while the CUPRAC method, that involves the use of a neutral medium has shown that the antioxidant activity of AgNP/NaLS was significantly improved compared to NaLS (*P* < 0.05). The lower values obtained by the DPPH method are probably explained by the steric inaccessibility of the radical due to the large molecular size of NaLS [96].

### 3.8 Biocompatibility

**Fig. 16:**
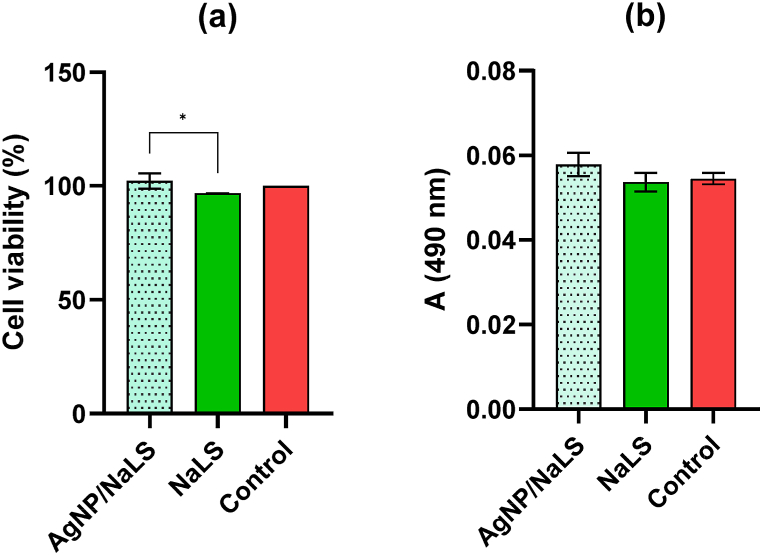
AgNP/NaLS and NaLS biocompatibility evaluation by the MTT (a) and LDH (b) tests under standard cultivation conditions of the HaCaT human keratinocyte cell line.

Both NaLS and AgNP/NaLS are biocompatible with the HaCaT cell line, the differences compared to the control being not statistically significant (Fig. 16a and b). However, NaLS was more cytotoxic than AgNP/NaLS (*P* < 0.05) (Fig. 16a), but no significant difference was observed in terms of LDH leakage (cell membrane integrity, *P* > 0.05) (Fig. 16b). Therefore, AgNP/NaLS proved to be biocompatible on the HaCaT cell line, as well as hemocompatible, so these NPs can be used in topical applications.

### 3.9 Antiproliferative effects on cal −27 cells

**Fig. 17:**
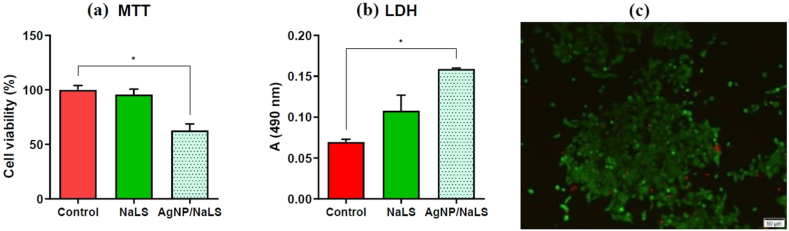
Cytotoxicity evaluation of AgNPs by MTT (a), LDH (b) and Live-Dead (c) assays under standard cultivation conditions of Cal −27 cells.

The MTT and LDH tests revealed that all tested variants have a cytotoxic effect on Cal-27 cells, demonstrated by reducing cell viability (as shown by the MTT test, Fig. 17a) and stimulating the extracellular release of LDH (Fig. 17b), an indicator of the cell death process. Corroboration of the results of the MTT and LDH tests indicates that the AgNP/NaLS solution presented a significant cytotoxic effect (*P* < 0.05), while NaLS is not cytotoxic. These results were also confirmed by the Live Dead assay (Fig. 17c).

## 4 Discussions

Synthesis methods and technologies related to green chemistry and nanosized materials are among the most rapidly growing fields of nowadays science. AgNPs synthesis methods in aqueous media are commonly performed in one or two stages, at room temperature [37,97], in hot, or boiling water [[98], [99], [100]]. In the case of the synthesis methods involving natural polysaccharides or polyphenolic chains used as both reductant and capping agents, reaction temperatures of 25 °C and 70 °C were frequently reported [101]. On the other hand, the time needed for an appropriate formation and growth of nanoparticles may differ with one or several orders of magnitude depending on both temperature and reaction environment. Considering these facts, the characteristics of one-pot AgNPs synthesis with NaLS have been examined for two key reaction temperatures: 25 °C (for one day) and 70 °C (for 2 h). The AgNPs were readily obtained at both temperatures by mixing the aqueous solutions of AgNO_3_ and NaLS, without the use of any other additive, surfactant or stabilizer except small amounts of NaOH required as co-reductant and to maintain a neutral pH.

The effects generated by the slow nucleation stage are supplementary enhanced by the solution behavior of NaLS. Nucleation and growth depend on the NaLS three-dimensional chain arrangement and flexibility in solution, which also influence the positioning and availability of its reactive groups. It was shown [51,53] that lignosulfonate macromolecules are prone to self-assembly and may form essentially flat aggregates by inter- and intramolecular π−π interactions between the aromatic rings. As a result, the charged groups are disposed on the surface and oriented toward the hydrophilic environment, which enhances the colloidal stability of NaLS. The heterogeneity of the reaction media also increases due to the NaLS micellar organization through aggregation at the edges of the polymer colloids. While the thickness of the aggregates remains unchanged, the other dimensions grow [53,80], affecting the process of AgNPs formation and maturation.

As previously shown, the process of AgNPs synthesis was significantly improved and developed much faster at 70 °C than at room temperature. Due to the fact that NaLS is stable at 70 °C, and no other changes were made, the potent increase in the AgNPs rate of nucleation and growth can be mainly explained by the fact that higher temperatures modify the solution behavior of NaLS. In essence, the temperature rise from 25 °C to 70 °C induces domino-type effects on the reaction mixture. The flexibility of three-dimensional polyphenolic chains increases, compromising the micellar self-assembly of NaLS. As a result, more phenolic hydroxyls became available for oxidation in a random fashion by silver ions, and more aromatic rings could initiate the adsorption of the cationic AgNPs seeds onto NaLS chains by cation-π noncovalent interactions [102]. The nucleation sites are multiplied and more chains show modified conformations due to the newly formed cation-π interactions. The dimensional growth is partially inhibited due to the physical cation-π interactions which enhance the steric hindrance provided by NaLS macromolecular chains. At the same time, the newly established interactions between AgNPs and NaLS prevent the reformation of typical NaLS micellar planar aggregates when colloidal solution is cooled to 25 °C and are strong enough to stabilize the nanoparticles against aggregation. The reduction of silver ions and capping interactions subsequently established between NaLS and newly formed AgNPs were furthermore detected in the infrared spectra, which confirm the results of UV–Vis data and solution behavior of lignosulfonates.

Since sodium lignosulfonate is basically a polyphenol, reductions take place at the level of phenolic hydroxyls through successive reactions involving the coordination of silver ions, electron transfer and deprotonation, with metallic silver and quinone formation [103,104], under the assistance of aqueous ionic hydroxyls.

The knowledge regarding lignosulfonate characteristics and aggregation behavior in aqueous solutions has significantly progressed in the last years [[51], [52], [53],^81^]. Hence, it has been reported that lignosulfonate concentration, counterions type and their distribution, are among the most important factors that guide self-assembly and micellization.

Visual observations evidenced that even diluted NaLS solutions start to flocculate after a couple of days to a few weeks, while AgNPs/NaLS dispersions form fine deposits after several weeks at room temperature, but these deposits could be easily resuspended by short mixing. These results are also important from the perspective of long-term preservation of AgNPs/NaLS mixtures, suggesting that fast desiccation under mixing applied to freshly prepared dispersions is a feasible method. The AgNPs lean towards aggregation in well separated nanoclusters associated with lignosulfonate-originated low contrast material, probably as result of sample preparation and water evaporation [47]. Nevertheless, the nanoparticles do not tend to fuse to each other even when are overlaid in agglomerations. In addition, the comparable sizes observed in solution and respectively in solid form are an indicative of good capped, relatively stable, and non-sticking nanoparticles, suggesting that desiccation of fresh AgNPs/NaLS solutions might represent a good solution for years long preservation. On the other hand, DLS results on raw systems obtained at 70 °C and stocked at 4 °C indicate a relative limited increase in sizes and polydispersity after more than one year. Such decays in AgNPs/NaLS dispersions should not impart a strong enough downside to halt their potential use. Thus, the systems obtained at 70 °C are stable during storage for sufficient time to allow their large-scale production and use in wound management. The result obtained strongly suggests that NaLS effectively capped *in situ* the newly formed AgNPs, restricting their growths, agglomeration and sedimentation, while the matured metallic core seems to mutually improve the solution stability of macromolecular chains. Furthermore, the comparable sizes obtained by several methods, both on solute and dried systems are a strong indicative that the overlaid NaLS is thin and tightly bound on the metallic surface, so it not substantially affects the hydrodynamic size of the system. The high negative values obtained for zeta potential also indicate that NaLS chains are oriented with acidic groups (mainly sulfonic) through the water interphase and phenol/quinone ones bonded at the level of metal interphase. In addition, these chains are actively protecting the agglomeration by the effect of steric hindrance induced by their three-dimensional, semi rigid structure.

Since XPS is essentially a surface analyzing technique that penetrates only to depths up to 10 nm [105], the combined effects of the nanoparticle sizes and capping interactions, NaLS solution characteristics, medium homogeneity, and dry film formation techniques could easily mask the changes brought to the polymer by silver ion reduction. For example, the slight increase in the C=C peak intensity (284.3 eV, NaLS; 285.0 eV, AgNPs/NaLS) is coupled with decreases in all other peaks associated with more flexible and hydrophilic lignosulfonate moieties in the presence of AgNPs. Such results could be explained by the orientation of AgNPs-bonded, rigid aromatic moieties at the contact layer with the hydrophobic air, while the hydrophilic parts try to remain in solution if the deposited film is not completely dry-out.

Wounds affect the skin structural integrity and its many crucial physiological roles, from sensorial, temperature control and homeostasis functions to protection against infections. Therefore, finding novel antimicrobial and wound healing promoting agents remains an urgent priority in the biomaterials research field. These agents should be active against different Gram-positive and Gram-negative bacteria that could infect the wounded skin and delay the healing process, especially if they form biofilms, defined as sessile microbial communities embedded in a self-secreted extracellular polymeric matrix, with high tolerance to conventional antimicrobial drugs. The most important bacterial species for wound care are *Staphylococcus aureus,* enterococci, *Pseudomonas aeruginosa* and Enterobacterales [106,107]. The antimicrobial activity is mainly due to the presence of Ag^+^, as NaLS showed only a weak activity. Our results are similar to those reported in other studies [108,109]. Also, the antimicrobial activity is influenced both by the size of the NPs and the chemical compoition of the extract or phenolic compounds used in their biosynthesis. The obtained results correlate with the literature data, related to AgNPs biosynthesized in compounds similar to sodium lignosulfonate. Thus, NPs biosynthesized in lignin extracted from dried *Acacia* wood dust (10–50 nm, dimensions similar to those obtained in this study) showed a weaker activity against *S. aureus* (6.0 ± 0.8 mm), but similar to *E. coli* (10.0 ± 2.3 mm) and *P. aeruginosa* (10.0 ± 0.8 mm) [110].

Several previous studies [[111], [112], [113], [114]] reported that the antibacterial performance of AgNPs is closely related to the fact that the free Ag^+^ released by AgNPs could interact with the cell wall and microbial membrane, increasing membrane permeability, disorganizing the cell structure or disrupting electron transport during ATP synthesis. The penetration of Ag^+^ into the cell wall can stimulate the formation of reactive oxygen species (ROS), inducing DNA damage and protein denaturation. Moreover, free Ag^+^ ions destabilize ribosomes in the cytoplasm and inhibit protein synthesis, thus leading to the destruction of metabolic activity and, finally, microbial death [115]. According to Alananbeh et al. [116], AgNPs derived from plant extracts with a larger size (more than 7 nm) can act as a continuous source of Ag^+^ release and facilitate prolonged antimicrobial activity during the application of AgNPs. Therefore, biosynthetic AgNPs could assure a remanent bacteriostasis exhibiting also the advantages of good biocompatibility and potential long-term stability.

The obtained MIC values are similar to those reported by Lintinen et al. [117], using carboxylate lignin particles as a biosynthesis system.

The AgNP/NaLS showed good antibacterial efficacy against both reference and wound infection strains. The results were expressed in terms of NaLS and Ag^+^. These nanoparticles demonstrated action, particularly against Gram negative bacteria involved in wound chronicity. Gram-positive bacteria, particularly *S. aureus*, appear to be the most common colonizers, particularly in the initial week of illness. Gram-negative bacteria, such as *P. aeruginosa* and *A. baumannii*, begin colonizing the wound around the beginning of the second week and can induce sepsis if they enter the lymphatic system or blood vessels [118]. When bacteria cells collect within a biofilm, antimicrobial dosages up to four times the MIC are necessary for biofilm eradication [119]. Biofilm is one of the trickiest elements in wound healing, with a prevalence rate of 60%–100% in chronic wounds. Biofilm has been detected in chronic leg ulcers, diabetic foot ulcers, pressure ulcers, burns, malignant and surgical wounds [118].

The microbial cells can sense NO in a concentration-dependent manner [120] and, in many bacterial species, NO can cause biofilm dispersal even at low concentrations (∼nM – μM) by triggering physiological responses primarily aimed at removing the NO from the cell [121]. Thus is very likely that such processes are activated in response to the presence of AgNPs.

NO can either be produced endogenously as a metabolic intermediate of denitrification or come from exogenous NO donors (such as AgNO_3_). The endogenously produced NO from bNOS has been shown to be critical for biofilm dispersal. Furthermore, when heterologously expressed, bNOS from *B. subtilis* has been shown to produce NO endogenously and enhance bacterial motility and decrease biofilm formation [[122], [123], [124]]. The NO may increase motility directly by acting on the production of flagella, pili, and/or rhamnolipids, or may indirectly stimulate nitrate reductase (NIR) expression to produce more NO. NIR expression or activity is also controlled by QS (RhlR and PQS), which thus modulates NO levels in response to population density. The response to NO in terms of biofilm formation/dispersion is, however, different under aerobic and anaerobic conditions, because anaerobiosis favors NO accumulation, ultimately leading to biofilm formation as a stress defense mechanism [125]. In addition, NO can modify many metabolic enzymes and other proteins responsible for basic physiological processes or virulence [126].

NO can either be produced endogenously as a metabolic intermediate of denitrification or come from exogenous NO donors (such as AgNO_3_). NO produced endogenously from bNOS has been shown to be critical for biofilm dispersal.

In higher concentrations, NO covalently binds DNA, proteins, and lipids, inducing oxidation and nitration, lipid peroxidation, mitochondrial dysfunction ultimately leading to cell death [127]. Thus, the extracellular accumulation of NO may be due to a pronounced denaturation of microbial proteins [128]. To confirm this hypothesis, four concentrations of AgNPs, including those corresponding to the MIC and MBC were evaluated at two different times (2 h and 4 h) regarding their effects on the Advanced Oxidation Protein Products (AOPP) levels, for which the NO_3_^−^ found in the reaction medium is generally responsible.

The role of NaLS in NPs is to generate antioxidant properties given by the phenolate, carboxylate and syringyl groups [129]. Green-synthesized AgNPs have an antioxidant potential comparable to NaLS. These findings showed that the presence of bioactive chemicals on the surface of AgNPs is responsible for the antioxidant activity and the silver nanoparticles do not contribute much, which is supported by previous research [130].

Also, according to Luna-Vázquez-Gómez et al. [131,132], AgNPs proved hemolytic at concentrations higher than 24 μg/mL of metallic silver, but the results obtained in this study by the presence of NaLS the hemolytic effect was reduced, probably due to the neutralization of free nitrogen and oxygen species given by the presence of NO^3−^ in the reaction medium [133]. Another study is showing that NaLS completely inhibited 2,2′-azobis (2-aminidopropane)-induced hemolysis in human blood at concentration of 200 mg/mL [129].

The hemo- and biocompatibility of these NPs was investigated in order to assess the possibility of progressing beyond the demonstration of functionality to their applicability. The hemolytic activity of most NPs is dependent on their concentration, structure, size and shape [134]. In the case of AgNPs, their hemolytic activity is mainly attributed to direct nanoparticle-cell interactions through thiol-type groups from the biological fragments, such as proteins and phospholipids in the erythrocyte membrane, leading to denaturation and impaired membrane function. Moreover, the negative charge of the functionalized AgNPs will have a stronger interaction with the biological cations in the erythrocyte membrane, leading to hemolysis [134,135].

To maintain its elasticity, structural and functional properties, the skin needs many substances, including antioxidant agents, with important and well-known functions such as: stimulating collagen synthesis and supporting antioxidant protection against UV-induced photo damage [136]. The development of effective antioxidant strategies based on target mechanisms seems to be a promising approach both in the prevention and therapy of human skin carcinogenesis and in the healing of chronic wounds, by regulating the redox balance at the wound level [137].

In the case of a skin trauma, the signaling cascade is triggered, starting with the activation of the immune system, with the infiltration of immune cells in the epidermis and dermis, followed by pro-inflammatory cytokines released by fibroblasts, keratinocytes and other immune cells. Concomitantly, hyperproliferation and inappropriate differentiation of keratinocytes and fibroblasts occur, switching to the wound healing phenotype [138]. Keratinocytes regulate wound healing through epithelial-mesenchymal interactions, having a critical role in wound repair as structural cells and exerting important immune functions [139]. Thus, it is very important that AgNP/NaLS to be non-cytotoxic to keratinocytes and to not disrupt their functions, namely the crosstalk between keratinocytes and immune cells involved in the healing of skin wounds [140].

While AgNP/NaLS system was virtually harmless on HaCaT human keratinocyte cells, it has been shown to exhibit a significant cytotoxic action against Cal −27 cells, suggesting another possible application of the synthesized nanoparticles, beyond the wound management, e.g., for the development of novel therapeutic strategies in specific skin cancers.

For the AgNPs obtained by the classical method, the concentrations of 10 μg/mL led to HaCaT cells viability between 30 and 60% [141] Due to the antioxidant properties of NaLS, a decrease of the cytotoxicity on HaCaT cells for AgNP/NaLS was observed, with a cellular viability of 102.17 ± 3.43% for a concentration of 13.7 μg/mL Ag^+^ [142].

Mitochondria are the cell's vital core [143], playing an important role in both physiological and pathological processes in cells, and are linked to cancer [144]. In squamous cell carcinoma (SCC), mitochondrial activities other than producing energy have been observed, including apoptosis induction, ROS formation, mitochondrial fission, and mitophagy [145]. However, it is worth mentioning that skin cells, by their nature, contain less mitochondria than tumor cells [146], such as SCC. Thus, cytotoxicity based on mitochondrial activity might be increased in cells with a high mitochondrial content. Mitochondria perform several functions in SCC; for example, mitochondrial calcium uniporter is substantially expressed in SCC compared to normal cells, and down-regulation of mitochondrial calcium uniporter has a significant impact on SCC cell proliferation and migration. An explanation for the decreased cytotoxicity induced by NaLS could be represented by its ability to perform ion exchange in aqueous medium *via* the formation of calcium-lignosulfonate (CaLS) [147]. The mitochondrial Ca^2+^ binding inhibits both mROS production and HIF-1 signaling pathway, which contribute to the tumor growth and metastasis formation [148].

## 5 Conclusions

A very simple, reliable, cost-efficient, and greener synthesis method to obtain stable and catalytically active AgNPs has been revisited, optimized and thoroughly evaluated as a potential source of bioactive products. Several experimental data, conditions, assumptions and reasoned hypothesis regarding the preparation of AgNPs/NaLS systems were not reported elsewhere, and could be of great interest from applicative point of view. The resulted AgNPs are polydisperse, with sizes ranging from 10 to 40 nm and only small variations due to temperature, initial ratios, and concentrations. It can be assumed that the NaLS is the key factor in obtaining AgNPs of appropriate sizes in a wide range of experimental conditions. As expected, despite the synthesis can be done without heating, the reaction temperature of 70 °C is the adequate one to have systems with desirable characteristics and stability. In addition, it was found that 1 mg of NaLS could efficiently reduce up to about 5 mmol of silver ions with subsequent formation of AgNPs, while polymer concentration should be lower than 12 mg/mL to avoid NaLS self-aggregation and nanoparticle agglomeration. The extensive characterization of AgNPs/NaLS proves that the proposed method is very effective and has translational potential into commercial products.

The AgNP/NaLS exhibited significant antibacterial activity, being effective especially against the problematic Gram-negative strains, both in planktonic (MIC values of 800–50 μg/mL expressed as NaLS concentration) and biofilm growth state (MBEC values of 800–50 μg/mL expressed as NaLS concentration). One possible mechanism of the antibiofilm effect of AgNP/NaLS might be the increase of NO release. At the same time, the protein oxidation is lower in bacterial cells treated with AgNPs functionalized with NaLS in comparison to AgNO_3_, suggesting a decreased toxicity of the hybrid colloids, e.g., against the normal human or environmental microbiota. The AgNP/NaLS proved to be non-hemolytic (2.26 ± 0.13% hemolysis) and biocompatible (102.17 ± 3.43 % HaCaT cells viability) at MIC and MBC values, supporting its use as an active principle for obtaining biomaterials intended for wound management. Moreover*,* the specificity of the cytotoxicity effects recorded on tumoral cells, but not on normal keratinocytes, might pave the way for other potential applications needing further confirmation such as skin cancer therapy.

## Ethics statement

This study was reviewed and approved by University of Bucharest Ethical Committee, with the approval number: 9/8121/October 02, 2018 (for isolated strains). This study was reviewed and approved by SC Sanimed International Impex SRL Ethical Committee, with the approval number: 3836/September 10, 2022 (for hemocompatibility). Review and/or approval by an ethics committee was not needed because the study did not involve testing on animals or human biological materials. Informed consent was not required for this study because we did not involve clinical testing on human subjects or human biological materials.

## Data availability statement

Data will be made available on request.

## CRediT authorship contribution statement

**Ioana C. Marinas:** Writing – review & editing, Writing – original draft, Validation, Software, Resources, Methodology, Investigation, Formal analysis, Data curation, Conceptualization. **Leonard Ignat:** Writing – review & editing, Writing – original draft, Validation, Software, Resources, Methodology, Investigation, Formal analysis, Data curation, Conceptualization. **Ignat E. Maurușa:** Writing – review & editing, Visualization, Methodology, Investigation, Formal analysis. **Madalina D. Gaboreanu:** Methodology, Investigation, Formal analysis. **Coroabă Adina:** Visualization, Methodology, Investigation, Formal analysis. **Marcela Popa:** Methodology, Investigation, Formal analysis. **Mariana C. Chifiriuc:** Writing – review & editing, Validation, Supervision, Funding acquisition, Conceptualization. **Marian Angheloiu:** Resources, Methodology. **Mihaela Georgescu:** Project administration, Methodology, Investigation, Funding acquisition, Formal analysis. **Alexandra Iacobescu:** Methodology, Investigation, Formal analysis. **Gratiela Gradisteanu Pircalabioru:** Methodology, Investigation, Formal analysis. **Miruna Stan:** Methodology, Investigation, Formal analysis. **Mariana Pinteala:** Writing – review & editing, Validation, Supervision, Conceptualization.

## Declaration of competing interest

The authors declare the following financial interests/personal relationships which may be considered as potential competing interests:Mariana Carmen Chifiriuc reports financial support was provided by Romanian 10.13039/501100005802National Authority for Scientific Research. Mihaela Georgescu reports financial support was provided by Romanian 10.13039/501100005802National Authority for Scientific Research.

## Abbreviations

AgNPs: Silver nanoparticles
AOPP: Advanced Oxidation Protein Products
ATCC: American Type Culture Collection
ATR-FTIR: Attenuated Total Reflectance - Fourier Transform Infrared Spectrometer
CFU: Colony forming unit
CUPRAC: Cupric reducing antioxidant capacity
DLS: Dynamic light scattering
DMEM: Dulbecco's Modified Eagle Medium
DPPH: 2,2-Diphenyl-1-picrylhydrazyl
EDTA: Ethylenediaminetetraacetic acid
FRAP: Ferric reducing antioxidant power
IDZ: Inhibition diameter zone
LDH: Lactate dehydrogenase
MBC: Minimum bactericidal concentration
MBEC: Microbial biofilm eradication concentration
MICs: Minimum inhibitory concentrations
MRSA: Methicillin-resistant *Staphylococcus aureus*
MTT: 3-(4,5-dimethylthiazol-2-yl)-2,5-diphenyltetrazolium
NaLS: Sodium lignosulphonate
NIR: Nitrate reductase
NO: Nitric oxide
PBS: Phosphate buffer saline
QS: Quorum sensing
RBC: Red blood cells
RNI: Reactive nitrogen intermediates
SPR: Surface plasmon resonance
TEM: Transmission electron microscope
TSB: Trypton Soy broth
XPS: X-ray photoelectron spectroscopy
XRD: X-ray diffractometer

## Footnotes

Appendix A Supplementary data to this article can be found online at https://doi.org/10.1016/j.heliyon.2024.e26047.

## References

[1] Asmatulu R. (2012).

[2] Johnston H.J., Hutchison G., Christensen F.M., Peters S., Hankin S., Stone V. (2010). A review of the in vivo and in vitro toxicity of silver and gold particulates: particle attributes and biological mechanisms responsible for the observed toxicity. Crit. Rev. Toxicol..

[3] Levard C., Hotze E.M., Lowry G.v., Brown G.E. (2012). Environmental transformations of silver nanoparticles: impact on stability and toxicity. Environ. Sci. Technol..

[4] Polshettiwar V., Varma R.S. (2010). Green chemistry by nano-catalysis. Green Chem..

[5] Yan N., Xiao C., Kou Y. (2010). Transition metal nanoparticle catalysis in green solvents. Coord. Chem. Rev..

[6] Hudson R., Feng Y., Varma R.S., Moores A. (2014). Bare magnetic nanoparticles: sustainable synthesis and applications in catalytic organic transformations. Green Chem..

[7] Anastas P.T., Warner J.C. (1998).

[8] Nowack B., Krug H.F., Height M. (2011). 120 Years of nanosilver history: implications for policy makers. Environ. Sci. Technol..

[9] Rai M., Yadav A., Gade A. (2009). Silver nanoparticles as a new generation of antimicrobials. Biotechnol. Adv..

[10] Sharma V.K., Yngard R.A., Lin Y. (2009). Silver nanoparticles: green synthesis and their antimicrobial activities. Adv. Colloid Interface Sci..

[11] dos Santos C.A., Seckler M.M., Ingle A.P., Gupta I., Galdiero S., Galdiero M. (2014). Silver nanoparticles: therapeutical uses, toxicity, and safety issues. J. Pharmaceut. Sci..

[12] Kokura S., Handa O., Takagi T., Ishikawa T., Naito Y., Yoshikawa T. (2010). Silver nanoparticles as a safe preservative for use in cosmetics. Nanomedicine.

[13] Siqueira M.C., Coelho G.F., de Moura M.R., Bresolin J.D., Hubinger S.Z., Marconcini J.M. (2014). Evaluation of antimicrobial activity of silver nanoparticles for carboxymethylcellulose film applications in food packaging. J. Nanosci. Nanotechnol..

[14] Perez D.P. (2010).

[15] Wen C., Yin A., Dai W.-L. (2014). Recent advances in silver-based heterogeneous catalysts for green chemistry processes. Appl. Catal., B.

[16] Chen L., Xie H., Li J. (2012). Electrochemical glucose biosensor based on silver nanoparticles/multiwalled carbon nanotubes modified electrode. J. Solid State Electrochem..

[17] Däwlätşina G.I., Minullina R.T., Fakhrullin R.F. (2013). Microworms swallow the nanobait: the use of nanocoated microbial cells for the direct delivery of nanoparticles into Caenorhabditis elegans. Nanoscale.

[18] Li H., Xu D. (2014). Silver nanoparticles as labels for applications in bioassays. TrAC Trends Anal. Chem. (Reference Ed.).

[19] Liu K., Qu S., Zhang X., Tan F., Wang Z. (2013). Improved photovoltaic performance of silicon nanowire/organic hybrid solar cells by incorporating silver nanoparticles. Nanoscale Res. Lett..

[20] Donnelly T., Smith W.E., Faulds K., Graham D. (2014). Silver and magnetic nanoparticles for sensitive DNA detection by SERS. Chem. Commun..

[21] Wiley B., Sun Y., Xia Y. (2007). Synthesis of silver nanostructures with controlled shapes and properties. Acc. Chem. Res..

[22] Christopher P., Linic S. (2010). Shape- and size-specific chemistry of Ag nanostructures in catalytic ethylene epoxidation. ChemCatChem.

[23] Banerjee S., Loza K., Meyer-Zaika W., Prymak O., Epple M. (2014). Structural evolution of silver nanoparticles during wet-chemical synthesis. Chem. Mater..

[24] Tsuji M., Gomi S., Maeda Y., Matsunaga M., Hikino S., Uto K. (2012). Rapid transformation from spherical nanoparticles, nanorods, cubes, or bipyramids to triangular prisms of silver with PVP, citrate, and H _2_ O _2_. Langmuir.

[25] Das D., Nath B.C., Phukon P., kalita A., Dolui S.K. (2013). Synthesis of ZnO nanoparticles and evaluation of antioxidant and cytotoxic activity. Colloids Surf. B Biointerfaces.

[26] Mari A., Imperatori P., Marchegiani G., Pilloni L., Mezzi A., Kaciulis S. (2010). High yield synthesis of pure alkanethiolate-capped silver nanoparticles. Langmuir.

[27] Virkutyte J., Varma R.S. (2011). Green synthesis of metal nanoparticles: biodegradable polymers and enzymes in stabilization and surface functionalization. Chem. Sci..

[28] Kim Y., Lee B.-G., Roh Y. (2013). Microbial synthesis of silver nanoparticles. J. Nanosci. Nanotechnol..

[29] Devi L.S., Joshi S.R. (2014). Evaluation of the antimicrobial potency of silver nanoparticles biosynthesized by using an endophytic fungus, Cryptosporiopsis ericae PS4. J. Microbiol..

[30] Hosseini-Abari A., Emtiazi G., Lee S.-H., Kim B.-G., Kim J.-H. (2014). Biosynthesis of silver nanoparticles by Bacillus stratosphericus spores and the role of dipicolinic acid in this process. Appl. Biochem. Biotechnol..

[31] Iravani S. (2011). Green synthesis of metal nanoparticles using plants. Green Chem..

[32] Akhtar M.S., Panwar J., Yun Y.-S. (2013). Biogenic synthesis of metallic nanoparticles by plant extracts. ACS Sustain. Chem. Eng..

[33] Borase H.P., Salunke B.K., Salunkhe R.B., Patil C.D., Hallsworth J.E., Kim B.S. (2014). Plant extract: a promising biomatrix for ecofriendly, controlled synthesis of silver nanoparticles. Appl. Biochem. Biotechnol..

[34] Park Y., Hong Y.N., Weyers A., Kim Y.S., Linhardt R.J. (2011). Polysaccharides and phytochemicals: a natural reservoir for the green synthesis of gold and silver nanoparticles. IET Nanobiotechnol..

[35] Pandey S., Goswami G.K., Nanda K.K. (2012). Green synthesis of biopolymer–silver nanoparticle nanocomposite: an optical sensor for ammonia detection. Int. J. Biol. Macromol..

[36] Kharissova O.v., Dias H.V.R., Kharisov B.I., Pérez B.O., Pérez V.M.J. (2013). The greener synthesis of nanoparticles. Trends Biotechnol..

[37] Nadagouda M.N., Varma R.S. (2008). Green synthesis of silver and palladium nanoparticles at room temperature using coffee and tea extract. Green Chem..

[38] Moulton M.C., Braydich-Stolle L.K., Nadagouda M.N., Kunzelman S., Hussain S.M., Varma R.S. (2010). Synthesis, characterization and biocompatibility of “green” synthesized silver nanoparticles using tea polyphenols. Nanoscale.

[39] Osorio-Román I.O., Ortega-Vásquez V., Vargas C.V., Aroca R.F. (2011). Surface-enhanced spectra on D-gluconic acid coated silver nanoparticles. Appl. Spectrosc..

[40] Black K.C.L., Liu Z., Messersmith P.B. (2011). Catechol redox induced formation of metal Core−Polymer shell nanoparticles. Chem. Mater..

[41] Peng H., Yang A., Xiong J. (2013). Green, microwave-assisted synthesis of silver nanoparticles using bamboo hemicelluloses and glucose in an aqueous medium. Carbohydr. Polym..

[42] Venkatesham M., Ayodhya D., Madhusudhan A., Veera Babu N., Veerabhadram G. (2014). A novel green one-step synthesis of silver nanoparticles using chitosan: catalytic activity and antimicrobial studies. Appl. Nanosci..

[43] Cheng F., Betts J.W., Kelly S.M., Schaller J., Heinze T. (2013). Synthesis and antibacterial effects of aqueous colloidal solutions of silver nanoparticles using aminocellulose as a combined reducing and capping reagent. Green Chem..

[44] Cao Y., Zheng R., Ji X., Liu H., Xie R., Yang W. (2014). Syntheses and characterization of nearly monodispersed, size-tunable silver nanoparticles over a wide size range of 7–200 nm by tannic acid reduction. Langmuir.

[45] Dubas S.T., Pimpan V. (2008). Humic acid assisted synthesis of silver nanoparticles and its application to herbicide detection. Mater. Lett..

[46] Coccia F., Tonucci L., Bosco D., Bressan M., d'Alessandro N. (2012). One-pot synthesis of lignin-stabilised platinum and palladium nanoparticles and their catalytic behaviour in oxidation and reduction reactions. Green Chem..

[47] Milczarek G., Rebis T., Fabianska J. (2013). One-step synthesis of lignosulfonate-stabilized silver nanoparticles. Colloids Surf. B Biointerfaces.

[48] Sixta H. (2006).

[49] Fredheim G.E., Christensen B.E. (2003). Polyelectrolyte complexes: interactions between lignosulfonate and chitosan. Biomacromolecules.

[50] Yang C., Liu P. (2009). Water-dispersed conductive polypyrroles doped with lignosulfonate and the weak temperature dependence of electrical conductivity. Ind. Eng. Chem. Res..

[51] Qiu X., Kong Q., Zhou M., Yang D. (2010). Aggregation behavior of sodium lignosulfonate in water solution. J. Phys. Chem. B.

[52] Deng Y., Feng X., Zhou M., Qian Y., Yu H., Qiu X. (2011). Investigation of aggregation and assembly of alkali lignin using iodine as a probe. Biomacromolecules.

[53] Vainio U., Lauten R.A., Haas S., Svedström K., Veiga L.S.I., Hoell A. (2012). Distribution of counterions around lignosulfonate macromolecules in different polar solvent mixtures. Langmuir.

[54] Ahmed I., Ready D., Wilson M., Knowles J.C. (2006). Antimicrobial effect of silver-doped phosphate-based glasses. J. Biomed. Mater. Res..

[55] Tarimala S., Kothari N., Abidi N., Hequet E., Fralick J., Dai L.L. (2006). New approach to antibacterial treatment of cotton fabric with silver nanoparticle–doped silica using sol–gel process. J. Appl. Polym. Sci..

[56] Arvinte A., Ignat M., Pinteala M., Ignat L. (2017). Electrochemical survey of silver nanoparticles-lignosulfonate formation and their assessment in the electrocatalytic oxidation of P-nitrophenol. Curr. Anal. Chem..

[57] Ignat L., Doroftei F., Ignat M.E., Iovan G., Grădinaru I. (2017). A facile and versatile procedure for deposition of silver nanoparticles on hydroxyapatite. EHB.

[58] Yang J., Liu L., An X., Seta F.T., Li C., Zhang H. (2021). Facile preparation of lignosulfonate induced silver nanoparticles for high efficient removal of organic contaminants in wastewater. Ind. Crop. Prod..

[59] Saratale R.G., Cho S.K., Saratale G.D., Kadam A.A., Ghodake G.S., Magotra V.K. (2022). Lignin-mediated silver nanoparticle synthesis for photocatalytic degradation of reactive yellow 4G and in vitro assessment of antioxidant, antidiabetic, and antibacterial activities. Polymers.

[60] Paladini F., Pollini M. (2019). Antimicrobial silver nanoparticles for wound healing application: progress and future trends. Materials.

[61] Rybka M., Ł Mazurek, Konop M. (2022). Beneficial effect of wound dressings containing silver and silver nanoparticles in wound healing—from experimental studies to clinical practice. Life.

[62] Vlad I.M., Nuță D.C., Ancuceanu R.V., Caproiou M.T., Dumitrascu F., Marinas I.C. (2021). New O-Aryl-Carbamoyl-Oxymino-Fluorene derivatives with MI-crobicidal and antibiofilm activity enhanced by combination with iron oxide nanoparticles. Molecules.

[63] Marinas I.C., Oprea E., Buleandra M., Badea I.A., Tihauan B.M., Marutescu L. (2021). Chemical composition, antipathogenic and cytotoxic activity of the essential oil extracted from amorpha fruticosa fruits. Molecules.

[64] Corbu V.M., Gheorghe-Barbu I., Marinas I.C., Avramescu S.M., Pecete I., Geanǎ E.I. (2022). Eco-friendly solution based on rosmarinus officinalis hydro-alcoholic extract to prevent biodeterioration of cultural heritage objects and buildings. Int. J. Mol. Sci..

[65] Quinteros M.A., Cano Aristizábal V., Dalmasso P.R., Paraje M.G., Páez P.L. (2016). Oxidative stress generation of silver nanoparticles in three bacterial genera and its relationship with the antimicrobial activity. Toxicol. Vitro.

[66] Braik A., Lahouel M., Merabet R., Djebar M.R., Morin D. (2019). Myocardial protection by propolis during prolonged hypothermic preservation. Cryobiology.

[67] Madhu G., Bose V.C., Aiswaryaraj A.S., Maniammal K., Biju V. (2013). Defect dependent antioxidant activity of nanostructured nickel oxide synthesized through a novel chemical method. Colloids Surf. A Physicochem. Eng. Asp..

[68] Thaipong K., Boonprakob U., Crosby K., Cisneros-Zevallos L., Hawkins Byrne D. (2006). Comparison of ABTS, DPPH, FRAP, and ORAC assays for estimating antioxidant activity from guava fruit extracts. J. Food Compos. Anal..

[69] Multescu M., Marinas I.C., Susman I.E., Belc N. (2022). Byproducts (flour, meals, and groats) from the vegetable oil industry as a potential source of antioxidants. Foods.

[70] Çelik S.E., Özyürek M., Güçlü K., Apak R. (2010). Determination of antioxidants by a novel on-line HPLC-cupric reducing antioxidant capacity (CUPRAC) assay with post-column detection. Anal. Chim. Acta.

[71] Njagi E.C., Huang H., Stafford L., Genuino H., Galindo H.M., Collins J.B. (2011). Biosynthesis of iron and silver nanoparticles at room temperature using aqueous sorghum bran extracts. Langmuir.

[72] Jayasmita J., Mainak G., Tarasankar P. (2016). Enlightening surface plasmon resonance effect of metal nanoparticles for practical spectroscopic application. RSC Adv..

[73] Qin Y., Ji X., Jing J., Liu H., Wu H., Yang W. (2010). Size control over spherical silver nanoparticles by ascorbic acid reduction. Colloids Surf. A Physicochem. Eng. Asp..

[74] Stevanović M., Savanović I., Uskoković V., Škapin S.D., Bračko I., Jovanović U. (2012). A new, simple, green, and one-pot four-component synthesis of bare and poly(α,γ,l-glutamic acid)-capped silver nanoparticles. Colloid Polym. Sci..

[75] Amendola V., Bakr O.M., Stellacci F.A. (2010). Study of the surface plasmon resonance of silver nanoparticles by the discrete dipole approximation method: effect of shape, size, structure, and assembly. Plasmonics.

[76] Jeong S.-H., Choi H., Kim J.Y., Lee T.-W. (2015). Silver-based nanoparticles for surface plasmon resonance in organic optoelectronics. Part. Part. Syst. Char..

[77] Rycenga M., Cobley C.M., Zeng J., Li W., Moran C.H., Zhang Q. (2011). Controlling the synthesis and assembly of silver nanostructures for plasmonic applications. Chem. Rev..

[78] Dong X., Ji X., Wu H., Zhao L., Li J., Yang W. (2009). Shape control of silver nanoparticles by stepwise citrate reduction. J. Phys. Chem. C.

[79] Nemes C.T., Vijapurapu D.K., Petoukhoff C.E., Cheung G.Z., O'Carroll D.M. (2013). Absorption and scattering effects by silver nanoparticles near the interface of organic/inorganic semiconductor tandem films. J. Nano Res..

[80] Deng Y., Wu Y., Qian Y., Ouyang X., Yang D., Qiu X. (2010). Adsorption and desorption behaviors of lignosulfonate during the self-assembly of multilayers. Bioresources.

[81] Ouyang X., Deng Y., Qian Y., Zhang P., Qiu X. (2011). Adsorption characteristics of lignosulfonates in salt-free and salt-added aqueous solutions. Biomacromolecules.

[82] Satheshkumar A., Ganesh K., Elango K.P. (2014). Charge transfer facilitated direct electrophilic substitution in phenylaminonaphthoquinones: experimental, theoretical and electrochemical studies. New J. Chem..

[83] Aguilar-Méndez M.A., San Martín-Martínez E., Ortega-Arroyo L., Cobián-Portillo G., Sánchez-Espíndola E. (2011). Synthesis and characterization of silver nanoparticles: effect on phytopathogen Colletotrichum gloesporioides. J. Nano Res..

[84] Shi Z., Xu G., Deng J., Dong M., Murugadoss V., Liu C. (2019). Structural characterization of lignin from *D. sinicus* by FTIR and NMR techniques. Green Chem. Lett. Rev..

[85] Li H., Liu H., Fu S., Zhan H. (2011). Surface hydrophobicity modification of cellulose fibers by layer-by-layer self-assembly of lignosulfonates. Bioresources.

[86] Kim S., Silva C., Zille A., Lopez C., Evtuguin D v, Cavaco-Paulo A. (2009). Characterisation of enzymatically oxidised lignosulfonates and their application on lignocellulosic fabrics. Polym. Int..

[87] Nath S., Kumar Ghosh S., Praharaj S., Panigrahi S., Basu S., Pal T. (2005). Silver organosol: synthesis, characterisation and localised surface plasmon resonance study. New J. Chem..

[88] Zhang N., Yu X., Hu J., Xue F., Ding E. (2013). Synthesis of silver nanoparticle-coated poly(styrene-co-sulfonic acid) hybrid materials and their application in surface-enhanced Raman scattering (SERS) tags. RSC Adv..

[89] Vigneshwaran N., Kathe A.A., Varadarajan P.V., Nachane R.P., Balasubramanya R.H. (2006). Biomimetics of silver nanoparticles by white rot fungus, Phaenerochaete chrysosporium. Colloids Surf., B.

[90] Liang M., Wang L., Su R., Qi W., Wang M., Yu Y. (2013). Synthesis of silver nanoparticles within cross-linked lysozyme crystals as recyclable catalysts for 4-nitrophenol reduction. Catal. Sci. Technol..

[91] You S.A., Kwon O.S., Jang J. (2012). A facile synthesis of uniform Ag nanoparticle decorated CVD-grown graphene via surface engineering. J. Mater. Chem..

[92] Agnihotri S., Mukherji S., Mukherji S. (2013). Immobilized silver nanoparticles enhance contact killing and show highest efficacy: elucidation of the mechanism of bactericidal action of silver. Nanoscale.

[93] Cai X., Lin M., Tan S., Mai W., Zhang Y., Liang Z. (2012). The use of polyethyleneimine-modified reduced graphene oxide as a substrate for silver nanoparticles to produce a material with lower cytotoxicity and long-term antibacterial activity. Carbon.

[94] Zheng J., Ding Y., Tian B., Wang Z.L., Zhuang X. (2008). Luminescent and Raman active silver nanoparticles with polycrystalline structure. J. Am. Chem. Soc..

[95] Quinteros M.A., Viviana C.A., Onnainty R., Mary V.S., Theumer M.G., Granero G.E. (2018). Biosynthesized silver nanoparticles: decoding their mechanism of action in Staphylococcus aureus and Escherichia coli. Int. J. Biochem. Cell Biol..

[96] Apak R., Güçlü K., Demirata B., Özyürek M., Çelik S., Bektaşoğlu B. (2007). Comparative evaluation of various total antioxidant capacity assays applied to phenolic compounds with the CUPRAC assay. Molecules.

[97] Pourjavadi A., Soleyman R. (2011). Silver nanoparticles with gelatin nanoshells: photochemical facile green synthesis and their antimicrobial activity. J. Nano Res..

[98] Bar H., Dkr Bhui, Sahoo G.P., Sarkar P., De S.P., Misra A. (2009). Green synthesis of silver nanoparticles using latex of Jatropha curcas. Colloids Surf., A.

[99] Kumar K.P., Paul W., Sharma C.P. (2012). Green synthesis of silver nanoparticles with zingiber officinale extract and study of its blood compatibility. Bionanoscience.

[100] Akaighe N., MacCuspie R.I., Navarro D.A., Aga D.S., Banerjee S., Sohn M. (2011). Humic acid-induced silver nanoparticle formation under environmentally relevant conditions. Environ. Sci. Technol..

[101] Bulut E., Özacar M. (2009). Rapid, facile synthesis of silver nanostructure using hydrolyzable tannin. Ind. Eng. Chem. Res..

[102] Pillai K.v., Renneckar S. (2009). Cation−π interactions as a mechanism in technical lignin adsorption to cationic surfaces. Biomacromolecules.

[103] Tarannum N., Bohra D., Gautam Y.K. (2019). Facile green synthesis and applications of silver nanoparticles: a state-of-the-art review. RSC Adv..

[104] Malik M.A., Batterjee M.G., Kamli M.R., Alzahrani K.A., Danish E.Y., Nabi A. (2022). Polyphenol-capped biogenic synthesis of noble metallic silver nanoparticles for antifungal activity against *Candida auris*. J Fungi.

[105] Li Z., Ge Y. (2011). Extraction of lignin from sugar cane bagasse and its modification into a high performance dispersant for pesticide formulations. J. Braz. Chem. Soc..

[106] Metcalf D.G., Bowler P.G. (2016). Clinician perceptions of wound biofilm. Int. Wound J..

[107] Rajkumari N., Mathur P., Misra M. (2014). Soft tissue and wound infections due to Enterococcus spp. among hospitalized trauma patients in a developing country. J. Global Infect. Dis..

[108] Verrillo M., Savy D., Cangemi S., Savarese C., Cozzolino V., Piccolo A. (2022). Valorization of lignins from energy crops and agro-industrial byproducts as antioxidant and antibacterial materials. J. Sci. Food Agric..

[109] Slavin Y.N., Ivanova K., Hoyo J., Perelshtein I., Owen G., Haegert A. (2021). Novel lignin-capped silver nanoparticles against multidrug-resistant bacteria. ACS Appl. Mater. Interfaces.

[110] Aadil K.R., Pandey N., Mussatto S.I., Jha H. (2019). Green synthesis of silver nanoparticles using acacia lignin, their cytotoxicity, catalytic, metal ion sensing capability and antibacterial activity. J. Environ. Chem. Eng..

[111] Pirtarighat S., Ghannadnia M., Baghshahi S. (2019). Green synthesis of silver nanoparticles using the plant extract of *Salvia spinosa* grown in vitro and their antibacterial activity assessment. J Nanostruct Chem.

[112] Kanniah P., Chelliah P., Thangapandi J.R., Gnanadhas G., Mahendran V., Robert M. (2021). Green synthesis of antibacterial and cytotoxic silver nanoparticles by *Piper nigrum* seed extract and development of antibacterial silver based chitosan nanocomposite. Int. J. Biol. Macromol..

[113] Musere S.F.P., Rahman A., Uahengo V., Naimhwaka J., Daniel L., Bhaskurani V.H.S., Jonnalagadda S.B. (2021). Biosynthesis of silver nanoparticles using pearl millet (*Pennisetum glaucum*) husk to remove algae in the water and catalytic oxidation of benzyl alcohol. J. Clean. Prod..

[114] Rather M.A., Deori P.J., Gupta K., Daimary N., Deka D., Qureshi A. (2022). Ecofriendly phytofabrication of silver nanoparticles using aqueous extract of *Cuphea carthagenensis* and their antioxidant potential and antibacterial activity against clinically important human pathogens. Chemosphere.

[115] Liu L., Yu C., Ahmad S., Ri C., Tang J. (2023). Preferential role of distinct phytochemicals in biosynthesis and antibacterial activity of silver nanoparticles. J. Environ. Manag..

[116] Alananbeh K.M., Al-Qudah Z., El-Adly A., Al Refaee W.J. (2017). Impact of silver nanoparticles on bacteria isolated from raw and treated wastewater in Madinah. KSA. Arab J Sci Eng.

[117] Lintinen K., Luiro S., Figueiredo P., Sakarinen E., Mousavi Z., Seitsonen J. (2019). Antimicrobial colloidal silver-lignin particles via ion and solvent exchange. ACS Sustain. Chem. Eng..

[118] Puca V., Marulli R.Z., Grande R., Vitale I., Niro A., Molinaro G. (2021). Microbial species isolated from infected wounds and antimicrobial resistance analysis: data emerging from a three-years retrospective study. Antibiotics.

[119] Grande R., Puca V., Muraro R. (2020). Antibiotic resistance and bacterial biofilm. Expert Opin. Ther. Pat..

[120] Lundberg J.O.N., Farkas-Szallasi T., Weitzberg E., Rinder J., Lidholm J., Änggåard A. (1995). High nitric oxide production in human paranasal sinuses. Nat. Med..

[121] Arora D.P., Hossain S., Xu Y., Boon E.M. (2015). Nitric oxide regulation of bacterial biofilms. Biochemistry.

[122] Yarullina D.R., Vakatova L.v., Krivoruchko A.v., Rubtsova E.v., Ilinskaya O.N. (2013). Effect of exogenous and endogenous nitric oxide on biofilm formation by Lactobacillus plantarum. Microbiology (N. Y.).

[123] Liu P., Huang Q., Chen W. (2012). Heterologous expression of bacterial nitric oxide synthase gene: a potential biological method to control biofilm development in the environment. Can. J. Microbiol..

[124] de Kievit T.R. (2009). Quorum sensing in *Pseudomonas aeruginosa* biofilms. Environ. Microbiol..

[125] Cutruzzolà F., Frankenberg-Dinkel N. (2016). Origin and impact of nitric oxide in Pseudomonas aeruginosa biofilms. J. Bacteriol..

[126] Urbano R., Karlinsey J.E., Libby S.J., Doulias P.-T., Ischiropoulos H., Warheit-Niemi H.I. (2018). Host nitric oxide disrupts microbial cell-to-cell communication to inhibit staphylococcal virulence. Cell Host Microbe.

[127] Radi R. (2018). Oxygen radicals, nitric oxide, and peroxynitrite: redox pathways in molecular medicine. P Natl Acad Sci.

[128] Schairer D.O., Chouake J.S., Nosanchuk J.D., Friedman A.J. (2012). The potential of nitric oxide releasing therapies as antimicrobial agents. Virulence.

[129] Aro T., Fatehi P. (2017). Production and application of lignosulfonates and sulfonated lignin. ChemSusChem.

[130] Bhutto A.A., Kalay Ş., Sherazi S.T.H., Culha M. (2018). Quantitative structure-activity relationship between antioxidant capacity of phenolic compounds and the plasmonic properties of silver nanoparticles. Talanta.

[131] Bedlovičová Z., Strapáč I., Baláž M., Salayová A. (2020). A brief overview on antioxidant activity determination of silver nanoparticles. Molecules.

[132] Luna-Vázquez-Gómez R., Arellano-García M.E., García-Ramos J.C., Radilla-Chávez P., Salas-Vargas D.S., Casillas-Figueroa F. (2021). Hemolysis of human erythrocytes by Argovit™ AgNPs from healthy and diabetic donors: an in vitro study. Materials.

[133] Quinteros M.A., Cano Aristizábal V., Dalmasso P.R., Paraje M.G., Páez P.L. (2016). Oxidative stress generation of silver nanoparticles in three bacterial genera and its relationship with the antimicrobial activity. Toxicol. Vitro.

[134] de la Harpe K., Kondiah P., Choonara Y., Marimuthu T., du Toit L., Pillay V. (2019). The hemocompatibility of nanoparticles: a review of cell–nanoparticle interactions and hemostasis. Cells.

[135] Das B., Tripathy S., Adhikary J., Chattopadhyay S., Mandal D., Dash S.K. (2017). Surface modification minimizes the toxicity of silver nanoparticles: an in vitro and in vivo study. J. Biol. Inorg. Chem..

[136] Capponi P.C., Murri D., Pernice C. (2021). Topical L-ascorbic acid formulation for a better management of non-melanoma skin cancer: perspective for treatment strategies. Pharmaceutics.

[137] Sander C.S., Chang H., Hamm F., Elsner P., Thiele J.J. (2004). Role of oxidative stress and the antioxidant network in cutaneous carcinogenesis. Int. J. Dermatol..

[138] Ricci A., Gallorini M., Feghali N., Sampò S., Cataldi A., Zara S. (2023). Snail slime extracted by a cruelty free method preserves viability and controls inflammation occurrence: a focus on fibroblasts. Molecules.

[139] Wong V.W., Akaishi S., Longaker M.T., Gurtner G.C. (2011). Pushing back: wound mechanotransduction in repair and regeneration. J. Invest. Dermatol..

[140] Piipponen M., Li D., Landén N.X. (2020). The immune functions of keratinocytes in skin wound healing. Int. J. Mol. Sci..

[141] Garvey M., Padmanabhan S., Pillai S. (2017). In vitro cytotoxicity of water soluble silver (Ag) nanoparticles on HaCat and A549 cell lines. J Toxicol Pharmacol.

[142] Zanette C., Pelin M., Crosera M., Adami G., Bovenzi M., Filon Larese F., Florio C. (2011). Silver nanoparticles exert a long-lasting antiproliferative effect on human keratinocyte HaCaT cell line. Toxicol. Vitro.

[143] Bai J., Wu L., Wang X., Wang Y., Shang Z., Jiang E., Shao Z. (2022). Roles of mitochondria in oral squamous cell carcinoma therapy: friend or foe?. Cancers.

[144] Porporato P.E., Filigheddu N., Bravo-San J.M., Kroemer G., Galluzzi L. (2018). Mitochondrial metabolism and cancer. Cell Res..

[145] Barba-Aliaga M., Alepuz P. (2022). Role of eIF5A in mitochondrial function. Int. J. Mol. Sci..

[146] Ilić K., Hartl S., Galić E., Tetyczka C., Pem B., Barbir R. (2021). Interaction of differently coated silver nanoparticles with skin and oral mucosal cells. J. Pharmaceut. Sci..

[147] Guo H., Sun P., Qin Z., Shan L., Zhang G., Cui S., Liang Y. (2014). Sodium lignosulfonate induced vaterite calcium carbonate with multilayered structure. Eur. J. Inorg. Chem..

[148] Tosatto A., Sommaggio R., Kummerow C., Bentham R.B., Blacker T.S., Berecz T. (2016). The mitochondrial calcium uniporter regulates breast cancer progression via HIF-1α. EMBO Mol. Med..

