# Supplementary material for: Insights into the physico-chemical and biological characterization of sodium lignosulfonate - silver nanosystems designed for wound management
Source: Heliyon. 2024 Feb 9;10(4):e26047. doi: 10.1016/j.heliyon.2024.e26047 (PMC10878957; doi:10.1016/j.heliyon.2024.e26047)
Supplement: Multimedia component 1 [file mmc1.docx]

**Supplementary Material**

Insights into the physico-chemical and biological characterization of sodium lignosulfonate - silver nanosystems designed for wound management

Ioana C. Marinas^1,2,†^, Leonard Ignat^3,†^ , Maurușa E. Ignat^3^, Madalina D. Gaboreanu^1,4^, Adina Coroabă^3^, Marcela Popa^1,4^, Mariana C. Chifiriuc ^1,4,5,6^, Marian Angheloiu^2^, Mihaela Georgescu^1^, Alexandra Iacobescu^3^, Gratiela Gradisteanu Pircalabioru^1,4^, Miruna Stan^1,7^, Mariana Pinteala^3^

^1^Research Institute of the University of Bucharest—ICUB, University of Bucharest, 050095 Bucharest, Romania; ^2^Research and Development Department of SC Sanimed International Impex SRL, 6 Bucharest -Giurgiu Street, 087040 Giurgiu, Romania;

^3^Centre of Advanced Research in Bionanoconjugates and Biopolymers, “Petru Poni” Institute of Macromolecular Chemistry, Gr. Ghica Voda Alley 41A, Iasi 700487, Romania;

^4^Faculty of Biology, Department of Botany and Microbiology, University of Bucharest, 1-3 Portocalelor Street, 060101, Bucharest, Romania;

^5^Romanian Academy of Scientists, 54 Spl. Independentei St., District 5, 50085 Bucharest, Romania;

^6^The Romanian Academy, 25, Calea Victoriei, Sector 1, District 1, 010071 Bucharest, Romania;

^7^Faculty of Biology, Department of Biochemistry and Molecular Biology, University of Bucharest, 91-95 Splaiul Independentei, 050095 Bucharest, Romania.

^†^These authors contributed to the work equally and should be regarded as co-first author.

^*^ These authors should be co-corresponding authors.

^*^Corresponding authors’ email: Leonard Ignat, lignat@icmpp.ro; Carmen M. Chifiriuc, carmen.chifiriuc@bio.unibuc.ro

**Fig. S1** Time evolution of maximum wavelength and absorbance for AgNPs/NaLS solutions during synthesis and storage at 25ºC.

**Fig. S2** Time evolution of maximum wavelength and absorbance for AgNPs/NaLS solutions during synthesis for 2 hours at 70ºC and later storage at 25ºC.







**Fig. S3** Zeta potential (left) and size distribution (right) of self-assembled NaLS colloidal micelles for solutions of 20 mg / mL and 2 mg / mL





**Fig. S4** XPS survey scan of NaLS.





**Fig. S5** XPS survey scan of AgNPs/NaLS synthesized at room temperature.





**Fig. S6** XPS survey scan of AgNPs/NaLS synthesized at 70ºC.










**Fig. S7** XPS spectra of O 1s for NaLS (a) and AgNPs synthesized at 25^0^C (b); 70^0^C (c).










**Fig. S8** XPS spectra of C 1s for NaLS (a) and AgNPs synthesized at 25ºC (b); 70ºC (c).










**Fig. S9** XPS spectra of S 2p for NaLS (a) and AgNPs synthesized at 25^0^C (b); 70^0^C (c).
